# Nonorthogonal Configuration Interaction of Constraint-Based Orbital-Optimized Excited States: A Versatile Method for Theoretical Photochemistry

**DOI:** 10.1021/acs.jctc.5c01064

**Published:** 2025-10-08

**Authors:** Yannick Lemke, Jörg Kussmann, Christian Ochsenfeld

**Affiliations:** † Chair of Theoretical Chemistry, Department of Chemistry, Ludwig-Maximilians-Universität München, D-81377 Munich, Germany; ‡ Max-Planck-Institute for Solid State Research, D-70569 Stuttgart, Germany

## Abstract

We introduce a nonorthogonal configuration interaction (NOCI) scheme for COOX, our recently developed constraint-based orbital-optimized excited state method [Kussmann et al. *J. Chem. Theory. Comput.*, **2024**, *20*, 8461], which enables a targeted variational optimization of electronically excited states through constrained density functional theory. COOX is shown to be a more reliable source of NOCI reference configurations compared to orbital-optimized methods based on the ΔSCF scheme. The versatility and stability of NOCI-COOX are illustrated for the 2 ^1^A_g_ state of all-*E*-polyenes, conical intersections, core excitations, and other cases that are challenging to traditional linear-response time-dependent DFT approaches, and exemplary calculations for the NOCI-COOX treatment of photoactive species in complex molecular or bulk environments by virtue of our recent e-COOX embedding scheme [Lemke et al. *Phys. Chem. Chem. Phys.*, **2025**, *27*, 12161] as well as polarizable continuum models are presented.

## Graphical abstract

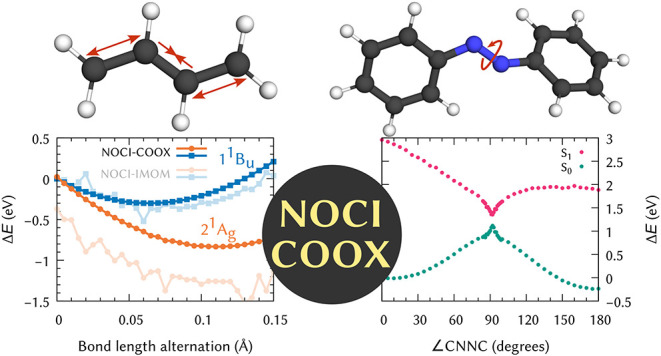

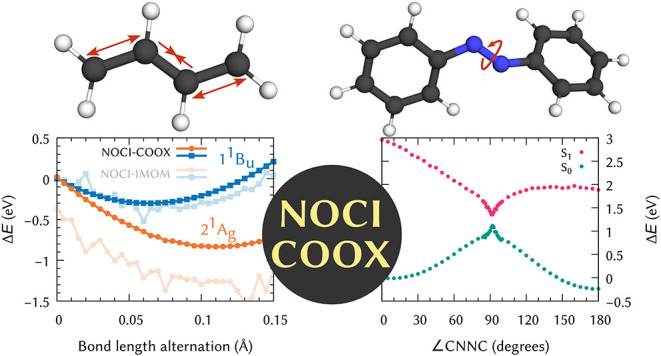

## 1 Introduction

The quantum-chemical study of electronically excited states, both in terms of vertical excitation energies and from a dynamics standpoint, is nowadays a ubiquitous and indispensable pillar in the design of novel photoactive compounds. While linear-response time-dependent density functional theory (LR-TDDFT) has been widely accepted by the quantum chemistry community as a decent compromise between accuracy and computational efficiency,^1–3^ its theoretical validity has been debated vividly^4–8^ and its numerous shortcomings are well-documented, e.g., for states with significant double-excitation character,^9,10^ charge-transfer states,^11–14^ core-excitations,^15,16^ and conical intersections.^17,18^ These inadequacies of LR-TDDFT, as well as the high computational cost and thus limited applicability of higher-level methods such as complete active space schemes or coupled-cluster-based approaches, have led to a recent resurgence of orbital-optimized methods, which enable the targeted computation of electronically excited states at the cost of a second self-consistent-field (SCF) procedure.

Orbital-optimized excited-state methods come in different flavors: The original ΔSCF approach^19–21^ and methods based thereupon such as the (initial) maximum overlap method (MOM/IMOM),^22,23^ state-targeted energy projection (STEP),^24^ restricted open-shell Kohn–Sham-DFT with squared gradient minimization (ROKS/SGM),^25^ or direct optimization with generalized mode-following (DO-GMF)^26,27^ variationally optimize stationary SCF solutions with non-Aufbau occupations, which offers a conceptually simple pathway to excited states, but is limited to well-defined single-orbital transitions and in some cases can suffer from poor convergence, convergence to an undesired state, or, as is often the case for the original formulation of ΔSCF, from variational collapse to the ground-state. When stable convergence can be achieved, ΔSCF-type methods have been shown to improve considerably upon LR-TDDFT for charge-transfer excitations,^28^ core-excitations,^29^ inverted-gap systems,^30,31^ and other challenging cases while maintaining overall good accuracy for small-radius Frenkel excitations.^32^ Another distinct advantage compared to linear-response methods is the direct applicability of a large subset of the ground-state toolkit to compute properties of interest such as molecular gradients, harmonic vibrational frequencies, etc. A noteworthy exception arising from the violation of the Aufbau principle is given by post-SCF correlation methods built upon the Laplace transform (e.g., efficient formulations of second-order Møller–Plesset perturbation theory^33–35^ or the random phase approximation^36^), which cannot be applied in the case of negative HOMO–LUMO gaps.

A second approach aiming to ensure a more stable convergence behavior is given by the “block-localized” methods of Gao and co-workers,^37–42^ which employ different subdivisions of the molecular orbital (MO) space into smaller, potentially nonorthogonal subspaces in which ΔSCF-type problems are solved, which lends itself nicely to long-range charge-transfer states, but can also be reformulated to handle local, small-radius Frenkel excitations.^39,40,42^

A third category of orbital-optimized methods is based on constrained density functional theory (cDFT),^43,44^ wherein excited states are optimized by enforcing additional constraints on the electron density. While the method was originally formulated with fragment-based charge or spin constraints in mind,^43,45,46^ several alternative constraints have been proposed as of late, e.g., constraints on the orthogonality between ground- and excited state,^47,48^ on the virtual orbital subspace,^49^ or on the LR-TDDFT transition density.^50^ Our recently proposed constraint-based orbital-optimized excited state method (COOX)^51^ also falls into the cDFT category; it employs a constraint on the static difference density, thereby recovering relaxation effects which would be missing in LR-TDDFT while maintaining the correct excited-state character. COOX has been shown to provide good accuracy across a broad spectrum of excited-state benchmark systems, including states with significant double-excitation character^51^ and core-excitations.^52^ Unlike most cDFT methods, the relaxed COOX density resembles a free oscillation of the unperturbed Hamiltonian, giving straightforward access to molecular gradients without the need for derivatives of the constraint potential and allowing the application of the previously mentioned Laplace-transform-based post-SCF correlation methods due to the adherence to the Aufbau principle.^51^ A straightforward embedding scheme for subsystem constraints has recently been used to extend the applicability of COOX to excited states in complex molecular or bulk environments in the full quantum system at reduced cost and improved accuracy.^53^

While there are several advantages of orbital-optimized methods over LR-TDDFT, a shared deficiency is the lack of static correlation in the single-reference mean-field picture, which can be incorporated by means of nonorthogonal configuration interaction (NOCI).^54–56^ While originally intended for use with Hartree–Fock determinants obtained from ΔSCF-type schemes,^55,56^ later modifications of NOCI were proposed to accommodate Kohn–Sham-DFT reference states^41,57^ as well as the inclusion of dynamic correlation by means of Møller–Plesset perturbation theory.^58,59^ A distinct advantage of NOCI over traditional (orthogonal) configuration interaction approaches is the issue of size-consistency: since the NOCI Hamiltonian is built from variationally relaxed SCF wave functions, which are themselves size-consistent, the NOCI wave function is, in principle, size-consistent as well.^55^ The matter becomes slightly more complicated if the underlying SCF solutions are spin-contaminated (as is often the case when using orbital-optimized methods), though spin rotation schemes have been developed to restore spin symmetry and size-consistency.^56,60^

In this work, based upon these preceding developments, we adapt our COOX method for the application in nonorthogonal configuration interaction. In the following Section 2, we provide a brief review of COOX and introduce the working equations for our NOCI-COOX ansatz, including the treatment of KS-DFT reference states. Section 3 briefly summarizes some implementation details as well as the computational setup used for the exploratory calculations, which we present in Section 4. These include challenging test cases, such as the bond length alternation (BLA) coordinate of butadiene, the pyramidalization of ethylene, and the cis–trans-isomerization of azobenzene. Section 5 concludes the paper and provides some pointers for potential avenues for future research.

## 2 Theory

Throughout this section, we will denote occupied and virtual MO indices by *i*, *j*,... and *a*, *b*,..., respectively, and atomic orbital (AO) indices by μ, ν,.... Orbital-optimized states are represented by Slater determinants |Φ⟩ and indexed by *I*, *J*,..., whereas correlated wave functions |Ψ⟩ are indexed by *n*, *m*,.... Furthermore, Einstein sum convention is explicitly *not* used throughout this work, so a term like *C*_μ*i*_ *C*_ν*i*_ represents the outer product of the *i*-th column vector of the MO coefficient matrix with itself rather than the contraction to an element of the density matrix.

### 2.1 Constraint-Based Orbital-Optimized Excitations

We begin with a brief recap of the COOX method for the variational relaxation of electronically excited states; for further details, we refer the reader to refs 51 and 52 .

The COOX method is rooted in the constrained DFT (cDFT) formalism;^44^ thus, excited states are optimized by enforcing additional constraints on the density

1 $$\int_{R^{3}}\rho^{\tau }\left(r\right)w_{c}^{\tau }\left(r\right)\;dr\equiv Tr\left[P^{\tau }W_{c}^{\tau }\right]\overset{!}{=}N_{c}$$

with the (spin-)­density ρ^τ^(**r**) with τ ∈ {α, β} and the corresponding one-particle density matrix **P** ^τ^, the constraint potential *w*_c_^τ^(**r**), which in matrix representation is expressed as *W*_c,μν_^τ^ = ⟨μ|*w*_c_^τ^|ν⟩, and the constraint target value *N*_c_. Inclusion in the Lagrange functional of the energy ensures that the constraint is satisfied:

2 $$Ẽ\left[\rho ,\lambda_{c};N_{c}\right]=E^{0}\left[\rho \right]+\lambda_{c}\left(Tr\left[PW_{c}\right]-N_{c}\right)$$

In COOX, the constraint potential is chosen as the static (i.e., unrelaxed) part of the difference density, typically obtained from a preceding LR-TDDFT calculation invoking the Tamm–Dancoff approximation (TDA)

3 $$W_{c}^{\tau }=S_{AO}\left(\Delta P^{virt,\tau }-\Delta P^{occ,\tau }\right)S_{AO}$$

with the AO overlap matrix **S**_AO,μν_ = ⟨μ|ν⟩ and the static difference densities

$$\Delta P_{\mu \nu }^{virt,\tau }=\underset{a,b}{\sum }C_{\mu a}^{\tau }\left(\underset{i}{\sum }X_{ai}^{\tau }X_{bi}^{\tau }\right)C_{\nu b}^{\tau }$$ 4

$$\Delta P_{\mu \nu }^{occ,\tau }=\underset{i,j}{\sum }C_{\mu i}^{\tau }\left(\underset{a}{\sum }X_{ai}^{\tau }X_{aj}^{\tau }\right)C_{\nu j}^{\tau }$$ 5

obtained from the MO coefficients **C** ^*τ*^ and the TDA-TDDFT transition amplitudes **X** ^*τ*^. For singlet excitations, we set **W**_c_^α^ = **W**_c_^β^, whereas for triplets, we set **W**_c_^α^ = −**W**_c_^β^. The constraint target value *N*_c_ is chosen such that the orbital spaces projected onto by Δ**P** ^virt^ and Δ**P** ^occ^ each contain one electron (i.e., a single excitation); therefore, we set *N*_c_ = 0. It should be stressed that COOX does not represent a spatial constraint in the typical sense associated with cDFT. While there is, of course, a spatial component to the constraint potential, in the sense that *w*_c_(**r**) can be interpreted as a multiplicative one-electron operator, the idea of spatial potentials in cDFT relates to the construction of *w*_c_(**r**) through real-space integration of fragment-based charge densities. In contrast, the COOX constraint represents an operator projecting onto parts of the occupied and virtual MO subspaces of the ground-state.

Typically, COOX singlet excitations would be optimized in a spin-restricted fashion using Fermi smearing to facilitate the equal population of Δ**P** ^occ^ and Δ**P** ^virt^ and to avoid spin-contamination. However, for the purposes of NOCI, integer occupation Slater determinants are required for the computation of wave function overlap and off-diagonal elements of the effective Hamiltonian. Therefore, in this work, we will be using COOX-optimized states not too dissimilar to those from ΔSCF approaches in which we enforce single spin orbital transitions. For example, consider a transition from φ_*i*_^α^ → φ_*a*_^α^, for which the corresponding difference densities are given by

$$\Delta P_{\mu \nu }^{virt}=\frac{1}{2}C_{\mu a}^{\alpha }C_{\nu a}^{\alpha }$$ 6

$$\Delta P_{\mu \nu }^{occ}=\frac{1}{2}C_{\mu i}^{\alpha }C_{\nu i}^{\alpha }$$ 7

which represents the outer product of MO coefficient matrix column vectors (note again that Einstein sum convention is explicitly *not* used). The excitation would leave orbital φ_*i*_^α^ unoccupied and φ_*a*_^α^ occupied, thus giving us a constraint target value of *N*_c_ = 1/2 due to the normalization factor of 1/2 in eqs 6 and 7. Due to the similarities shared with ΔSCF, we term this ansatz “ΔCOOX.” While we predominantly use the ΔCOOX wave functions to construct our NOCI Hamiltonian, one could of course also compute vertical excitations directly from the ΔCOOX-SCF energies. This approach, along with comparisons to ΔSCF and related methods, will be investigated in future work. Since eqs 6 and 7 only constrain the α-spin sector, the resulting orbital-optimized state would be severely spin-contaminated; we therefore also include its spin-flipped version in the NOCI Hamiltonian for the purposes of spin-purification. It should be noted that this approach only provides partial purification, as a complete purification procedure would require a combinatorial number of determinants per reference state to be included in the Hamiltonian.^61^ A more cost-effective middle-ground could be given by unitary rotations with discretized angles θ between the α- and β-spin sectors^56,60^ (of which our chosen approach is a special case with two angles θ_1_ = 0° and θ_2_ = 90°); however, such approaches were not deemed necessary for the present work as discussed in Section 3. We further include double-excitations within our NOCI expansion, which can generally be obtained through the (spin-independent) difference densities

$$\Delta P_{\mu \nu }^{virt}=\frac{1}{2}\left(C_{\mu a}C_{\nu a}+C_{\mu b}C_{\nu b}\right)$$ 8

$$\Delta P_{\mu \nu }^{occ}=\frac{1}{2}\left(C_{\mu i}C_{\nu i}+C_{\mu j}C_{\nu j}\right)$$ 9

for some occupied orbitals *i* and *j* and virtual orbitals *a* and *b*, with a constraint target value of *N*_c_ = 1. For the purposes of computational feasibility, we limit our calculations to double-excitations of seniority zero (*i* = *j*, *a* = *b*) and two (*i* = *j*, *a* ≠ *b* or *i* ≠ *j*, *a* = *b*).

At this point, it is instructive to recapitulate how COOX differs from other orbital-optimized DFT approaches. Considering other flavors of cDFT, COOX offers access to arbitrary excited states without a growing number of constraints in contrast to the x-cDFT method of Ramos and Pavanello^49^ and, unlike the t-cDFT approach of Stella et al.,^50^ yields physically correct excited-state densities and related properties. While in this work we limit our calculations to single-orbital transitions, in general, COOX and some other cDFT approaches like the orthogonality-constrained method of Pham and Khaliullin^48^ are by design not limited to clear-cut single-orbital transitions, which are always required in schemes based on the ΔSCF procedure^19–21^ such as (I)­MOM,^22,23^ STEP,^24^ and ROKS/SGM.^25^ Unlike ΔSCF, COOX is not prone to variational collapse due to non-Aufbau occupations, since the population of the virtual space is not achieved through manual exchange of orbitals in the construction of the density matrix, but rather variationally through the Lagrange functional and optimization of the respective Lagrange multiplier λ_c_. Naturally, this approach adds computational overhead due to repeated diagonalizations of the perturbed Fock matrix to determine λ_c_; however, in the case of single constraints (as is the case in COOX), efficient bracketing algorithms can yield λ_c_ to satisfactory accuracy of |Tr­[**PW**_c_] – *N*_c_| < 10^–10^ within a few (<10) iterations. Furthermore, in our experience, COOX exhibits a better algorithmic stability with common convergence acceleration techniques like Pulay’s direct inversion of the iterative subspace,^62,63^ thus requiring fewer total SCF iterations (and therefore fewer Fock builds) to achieve convergence, which ameliorates the added cost of repeated diagonalization to some degree. It should be noted that the development of novel algorithms for ΔSCF is still a highly active field of research, and recent developments such as SGM^25^ or DO-GMF^27^ have generally led to improved stability for ΔSCF methods, albeit at the expense of generally higher computational cost and a larger number of required SCF iterations. Finally, the block-localized excitation methods of Gao and co-workers^39,40,42^ achieve orbital-optimized excited states by solving ΔSCF-type problems in predefined subspaces. In particular, the target state-optimized DFT (TSO-DFT) approach^42^ seems to offer vast flexibility for the definition of these subspaces, which should in theory enable the application to mixed excitation patterns, though to our knowledge, such cases have not been investigated in the literature yet. Furthermore, for sensibly chosen subspaces, variational collapse can be avoided; nevertheless, we argue that this approach requires considerably more manual intervention than the simple COOX ansatz.

### 2.2 Nonorthogonal Configuration Interaction

We now turn our attention to the formulation of the relevant NOCI-COOX equations. In general, CI approaches produce correlated wave functions through the diagonalization of an effective Hamiltonian, which amounts to solving the secular equation

10 HC=SCE

Here,

11 $$H_{IJ}=\left\langle \Phi_{I}\left|Ĥ\right|\Phi_{J}\right\rangle$$

is a matrix element of the effective Hamiltonian evaluated using Slater determinants ⟨Φ_*I*_| and |Φ_*J*_⟩

12 $$S_{IJ}=\left\langle \Phi_{I}|\Phi_{J}\right\rangle$$

is an element of the generally nondiagonal overlap matrix, and C and E are the unknown CI expansion coefficients and energies, respectively. We solve eq 10 in a similar fashion to the well-known Roothaan–Hall equations through Löwdin orthogonalization^64^ and subsequent diagonalization of H (in the case of linear dependencies, canonical orthogonalization^65^ is used instead). From C , other important quantities can be computed, such as the spin expectation value

13 $${\left\langle {Ŝ}^{2}\right\rangle }_{n}=\left\langle \Psi_{n}\left|{Ŝ}^{2}\right|\Psi_{n}\right\rangle =\sum_{IJ}C_{I}^{n}C_{J}^{n}\left\langle \Phi_{I}\left|{Ŝ}^{2}\right|\Phi_{J}\right\rangle$$

(see Appendix A for a detailed derivation) and the transition density matrix

15 γnm(r,r′)=∑IJCInCJmγIJ(r,r′)(14)=∑IJCInCJm∑pqτφ̃pτI(r)φ̃qτJ(r′)⟨ΦI|âpτ†âqτ|ΦJ⟩(15)

where φ̃pτI are transformed molecular spin orbitals belonging to the determinant Φ_*I*_, which shall be explained in more detail below, and ${â}_{p\tau }^{†}$ and ${â}_{q\tau }$ are the usual particle creation and annihilation operators. Of particular interest are the transition densities ^*n*0^ *γ*, from which transition dipole moments and oscillator strengths between the correlated ground-state and excited states can be computed, giving rise to simulated spectra.

### 2.3 Hartree–Fock Reference States

To formulate our NOCI Hamiltonian in the Hartree–Fock case, we adopt the formalism of Head-Gordon and co-workers,^55,56^ wherein the reference determinants |Φ_*I*_⟩, |Φ_*J*_⟩, ... are transformed into a pairwise biorthogonal basis. More specifically, we follow the approach of Sundstrom and Head-Gordon,^56^ which employs a singular value decomposition (SVD) of the MO overlap matrix

SMOIJ=(CoccI)TSAO(CoccJ)=UΣVT 16

where ^*I*^ **C**_occ_ and ^*J*^ **C**_occ_ are the occupied MO coefficients of |Φ_*I*_⟩ and |Φ_*J*_⟩, respectively, **U** and **V** are orthogonal matrices, and **Σ** is a rectangular (in this case square) diagonal matrix with non-negative, decreasingly ordered diagonal entries Σ_*ii*_ ≡ σ_*i*_ ≥ 0, σ_*i*_ ≥ σ_*i*+1_. The overlap between the Slater determinants is then given by

17 ⟨ΦI|ΦJ⟩=det(SMOIJ)=±∏i=1Noccσi=±∏i=1Nασiα∏j=1Nβσjβ

where the phase factor is dictated by the sign of det­(^*IJ*^ **S**_MO_) or equivalently det­(**U**)­det­(**V** ^T^). The transformed coefficient matrices, which are used for the computation of the off-diagonal Hamiltonian elements, are given by

18 C̃occI=CoccIU

19 C̃occJ=CoccJV

respectively.

For Hartree–Fock reference determinants, the required integrals for the Hamiltonian can be derived through generalized Slater–Condon rules.^66^ In the AO basis, these require weighted codensity matrices

20 PμντIJ=∑i=1NτC̃μiτIC̃νiτJσiτ

21 PIJ=PαIJ+PβIJ

as well as dyadic density matrices

22 Di,μντIJ=C̃μiτIC̃νiτJ

The resulting integrals are the ones derived by Thom and Head-Gordon,^55^ which we list in Table 1 for completeness. Note in particular that, since the singular values **σ** ^α^ and **σ** ^β^ are non-negative and decreasing, any zeros in **σ** ^α^ and **σ** ^β^ must appear as their last elements. With respect to the system size *M*, the construction of H formally scales as *O*(*M* ^3^) per determinant pair due to the required SVD, though at practical system sizes (up to a few hundred atoms), the computation of the exchange matrix **K**, which formally scales as *O*(*M* ^2^) for standard algorithms, represents the most time-consuming step. The overall cost of the method can therefore be summarized as *O*(*N*_Φ_^2^ *M* ^3^), where *N*_Φ_ is the number of nonorthogonal determinants included in the NOCI expansion.

**Table 1:** Off-Diagonal Hamiltonian Elements $\left\langle \Phi_{I}\left|Ĥ\right|\Phi_{J}\right\rangle$ in a Biorthogonal Representation Obtained through Singular Value Decomposition^a^

zeros in **σ**	expression for $\left\langle \Phi_{I}\left|Ĥ\right|\Phi_{J}\right\rangle$
none	±∏i=1Nασiα∏j=1Nβσjβ(Tr{PIJh}+12(Tr{PIJJ[PIJ]}−Tr{PαK[PαIJ]IJ}−Tr{PβK[PβIJ]IJ})+VNN)
$\sigma_{N_{\alpha }}^{\alpha }$	±∏i=1Nα−1σiα∏j=1Nβσjβ(Tr{DNααIJh}+12(Tr{DNααIJJ[PIJ]}−Tr{DNααIJK[PαIJ]}))
$$\sigma_{N_{\beta }}^{\beta }$$	analogous to $\sigma_{N_{\alpha }}^{\alpha }$
$\sigma_{N_{\alpha }-1}^{\alpha },\;\sigma_{N_{\alpha }}^{\alpha }$	±12∏i=1Nα−2σiα∏j=1Nβσjβ(Tr{DNα−1αIJJ[DNααIJ]}−Tr{DNα−1αIJK[DNααIJ]})
$$\sigma_{N_{\beta }-1}^{\beta },\;\sigma_{N_{\beta }}^{\beta }$$	analogous to $\sigma_{N_{\alpha }-1}^{\alpha },\sigma_{N_{\alpha }}^{\alpha }$
$\sigma_{N_{\alpha }}^{\alpha }{,\;\sigma }_{N_{\beta }}^{\beta }$	±12∏i=1Nα−1σiα∏j=1Nβ−1σjβTr{DNααIJJ[DNββIJ]}
otherwise	0

a The matrix **h** denotes the core Hamiltonian, and **J**[**P**] and **K**[**P**] denote Coulomb and exchange matrices constructed from a density matrix **P**, respectively. The definitions for the weighted and dyadic codensity matrices are given in eqs 20–22. *V*_NN_ is the nuclear–nuclear repulsion potential.

Since we use spin-contaminated reference determinants, the computation of $\left\langle {Ŝ}^{2}\right\rangle$ for the final NOCI wave functions is crucial to verify that they are (approximate) eigenfunctions of ${Ŝ}^{2}$ . In ref 56, Sundstrom and Head-Gordon gave expressions for integrals $\left\langle \Phi_{I}\left|{Ŝ}^{2}\right|\Phi_{J}\right\rangle$ derived from the pairwise biorthogonal basis in the context of Generalized Hartree–Fock (GHF) theory, which employed a resolution-of-the-identity approach with all singly excited determinants which can be constructed from ⟨Φ_*I*_| and |Φ_*J*_⟩. In the context of regular unrestricted Hartree–Fock (UHF) or Kohn–Sham (UKS) theory, these terms simplify drastically, as we show in Appendix A.

### 2.4 Kohn–Sham-DFT Reference States

The design of an appropriate Hamiltonian in the case of Kohn–Sham-DFT reference states is somewhat more challenging, since we know neither the form of the wave function of the interacting system nor the expressions for the corresponding Hamiltonian matrix elements. The first issue is usually circumvented by using the Slater determinant of the noninteracting system in place of the correlated wave function. To address the second issue, a number of approaches have been discussed in the literature: Van Voorhis and co-workers have proposed an effective Hamiltonian specially designed for cDFT which interprets the Kohn–Sham determinants as approximate eigenfunctions of the perturbed Hamiltonian^57^

23 (ĤDFT+λcIŴcI)|ΦI⟩≈(EKS[ρI]+λcI⁡NcI)|ΦI⟩

where ŴcI , *^I^N*_c_, and ^*I*^λ_c_ are the constraint potential, target value, and optimized Lagrange multiplier for state *I*, respectively. Thus, exploiting the hermiticity of ${Ĥ}^{DFT}$ , the effective NOCI Hamiltonian can be expressed as

25 ⟨ΦI|ĤDFT|ΦJ⟩=12[⟨ΦI|ĤDFT+λcIŴcI−λcIŴcI|ΦJ⟩+⟨ΦI|ĤDFT+λcJŴcJ−λcJŴcJ|ΦJ⟩](24)≈⟨ΦI|ΦJ⟩2[EKS[ρI]+λcI⁡NcI+EKS[ρJ]+λcJ⁡NcJ]−12⟨ΦI|λcIŴcI+λcJŴcJ|ΦJ⟩(25)

Such a representation is particularly convenient since it requires no additional evaluation of costly two-electron integrals but has been found impractical for our purposes: As previously mentioned, we will, in general, be dealing with spin-contaminated determinants if we enforce a single-orbital transition, and any spin-purification should be accomplished through diagonalization of the effective Hamiltonian. However, the Hamiltonian, as expressed in eq 25, does not provide the necessary purification if the relaxed excited-state determinant, say |Φ_*I*_⟩, and its spin-flipped counterpart $|\overset{®}{\Phi_{I}}\rangle$ are (nearly) orthogonal. In this case, |Φ_*I*_⟩ and $|\overset{®}{\Phi_{I}}\rangle$ will differ in at least two orbitals due to spin symmetry (or analogously, the singular values **σ** ^α^ and **σ** ^β^ will contain at least one zero entry each). The first term in eq 25 clearly vanishes if $\left\langle \Phi_{I}|\overset{®}{\Phi_{I}}\right\rangle =0$ , whereas the second term vanishes because it only contains one-electron integrals, but the wave functions differ in at least two orbitals. Therefore, the coupling element between the two states is zero, and the resulting spin-adapted singlet and triplet linear combinations have identical energies.

Alternatively, a semiempirical approach similar to the popular density functional theory and multireference configuration interaction (DFT/MRCI) method^67,68^ could be a promising direction; however, combining nonorthogonal orbital-optimized reference states into well-defined configuration state functions may prove challenging.

A third possibility, which we shall use for our NOCI-COOX Hamiltonian, is given by the multistate DFT method (MS-DFT) of Gao and co-workers,^37–41^ wherein the DFT Hamiltonian is expressed in terms of the regular molecular Hamiltonian Ĥ and an unknown transition density functional^39^

26 ⟨ΦI|ĤDFT|ΦJ⟩=⟨ΦI|Ĥ|ΦJ⟩+ETDF[γIJ,ΦI,ΦJ]

where ^*IJ*^γ is the transition density matrix between states *I* and *J*, which in MS-DFT are typically represented by nonorthogonal, block-localized diabatic states, but in our case will be given by relaxed ΔCOOX determinants. The term $\left\langle \Phi_{I}\left|Ĥ\right|\Phi_{J}\right\rangle$ can once again be evaluated using the biorthogonal representation and the resulting expressions summarized in Table 1. For *E* ^TDF^, Gao and co-workers proposed two simple transition-density-independent approximations, i.e., a “determinant-weighted” average of the KS correlation corrections^38^

27 $$E^{TDF}\left[\Phi_{I},\Phi_{J}\right]\approx \frac{\left\langle \Phi_{I}\left|Ĥ\right|\Phi_{J}\right\rangle }{E^{HF}\left[\Phi_{I}\right]+E^{HF}\left[\Phi_{J}\right]}\left(\Delta E_{c}^{KS}\left[\Phi_{I}\right]+\Delta E_{c}^{KS}\left[\Phi_{J}\right]\right)$$

and an “overlap-weighted” average of the KS correlation corrections^37^

28 $$E^{TDF}\left[\Phi_{I},\Phi_{J}\right]\approx \frac{\left\langle \Phi_{I}|\Phi_{J}\right\rangle }{2}\left(\Delta E_{c}^{KS}\left[\Phi_{I}\right]+\Delta E_{c}^{KS}\left[\Phi_{J}\right]\right)$$

Note that Δ*E*_c_^KS^ in eqs 27 and 28 is *not* the regular KS correlation functional, but rather the difference between the KS and HF energies evaluated using the KS determinant Φ_*I*_ (and corresponding density ρ_*I*_)­

29 $$\Delta E_{c}^{KS}\left[\Phi_{I}\right]=E^{KS}\left[\rho_{I}\right]-E^{HF}\left[\Phi_{I}\right]$$

We have found the numerical differences between both approaches to be small for NOCI-COOX; throughout this work, we shall therefore adopt the determinant-weighted correction as formulated in eq 27.

## 3 Computational Details

The described NOCI scheme has been implemented in our FermiONs++ program package^69–71^ for both ΔCOOX and ΔSCF-type reference states. Tight thresholds for integral screening (ϑ_int_ = 10^–10^ a.u.) and SCF convergence (ϑ_RMS([**F**,**P**])_ = 10^–7^ a.u.) were applied throughout. Unless stated otherwise, the def2-TZVP basis set^72^ was used in all calculations. For the computation of exact exchange (EXX), we used the sn-LinK method^73,74^ with a g4 grid,^75^ and the LIBXC library^76,77^ was used to compute exchange–correlation integrals in Kohn–Sham-DFT calculations, where a gm5 grid^75^ was employed. Where necessary, geometry optimizations were carried out at the B3LYP-D3­(BJ)/def2-TZVP level of theory^78–80^ using the simple-dftd3 library^81^ for dispersion corrections; all relevant geometries are provided in the Supporting Information. We have found that density fitting for Coulomb integrals can lead to numerical instabilities in the NOCI procedure; therefore, Coulomb integrals are evaluated analytically using the regular J-engine.^82,83^ For NOCI, we consider transformed MOs φ̃iτI and φ̃iτJ to be orthogonal if their corresponding singular value σ_*i*_^τ^ is less than 10^–6^. All timings were recorded on a single compute node with two AMD EPYC 7452 processors (in total, 64 cores, 128 threads) and 1 TiB of DDR4 memory clocked at 3200 MT/s. As stated before, we perform broken-symmetry ΔCOOX calculations to obtain orbital-optimized reference states and include both spin configurations of spin-contaminated states for the purposes of purification. If our NOCI Hamiltonian contained only one such state, this would be reminiscent of the half-projected Hartree–Fock (HPHF) method of Smeyers et al.,^84,85^ with the distinction being that we perform spin-projection on variationally optimized wave functions rather than variationally optimizing a spin-projected wave function. This approach was found sufficient for most systems investigated in this work, though more sophisticated spin-purification schemes^56,60,61^ might be of interest for future applications. Since the computation of EXX in the NOCI Hamiltonian (which is always required irrespective of the underlying Kohn–Sham functional) is by far the most time-consuming step, we will predominantly be using hybrid functionals for the generation of orbital-optimized reference states, namely, the PBE0 (PBEh) functional with 25% EXX^86,87^ and the BHandHLYP functional with 50% EXX,^88^ which the DFT/MRCI Hamiltonian is optimized for.^67,68^

One remaining variable is the reference states to be included in the construction of the NOCI Hamiltonian. Naturally, for simple systems, these can be selected manually, though a more black-box approach is desirable. To that extent, we employ a kind of “active space” scheme similar to that in DFT/MRCI,^67,68^ wherein we only include transitions whose orbital energy differences fall within a predetermined threshold

30 $$\sum_{a\in exc}^{virt}\varepsilon_{a}-\sum_{i\in exc}^{occ}\varepsilon_{i}\leq \Delta \varepsilon_{\max}$$

where “*a* ∈ exc” and “*i* ∈ exc” denote all virtual and occupied orbitals partaking in the formation of the excitation constraint **W**_c_, respectively. Optionally, one can further limit the excitation space to a user-defined subset of occupied and virtual orbitals.

## 4 Results and Discussion

### 4.1 Low-Lying Singlet States of Polyenes

The low-lying 1 ^1^B_u_ and 2 ^1^A_g_ states of all-*E*-polyenes C_2*n*_H_2*n*+2_ have been the subject of countless theoretical studies. The experimentally dark 2 ^1^A_g_ state, in particular, poses a considerable challenge to excited-state methods due to its partial double-excitation character. For example, while LR-TDDFT yields acceptable excitation energies, the resulting excited-state density and related properties are physically incorrect—for instance, LR-TDDFT does not provide an accurate description of the BLA “breathing mode” of butadiene.^17^ In a previous work, we have shown that, using a scaled version of the COOX constraint, a physically correct 2 ^1^A_g_ excited-state density at the reference geometry can be obtained while maintaining the partial double-excitation character.^51^ Therefore, all-*E*-polyenes represent an ideal test system for the proposed NOCI-COOX scheme. For the sake of simplicity, we initially use a small manual excitation space also adopted by Sundstrom and Head-Gordon,^56^ which we list in Table 2. Notably, ΔCOOX as defined in eqs 8 and 9 yields spin-restricted states for the seniority-two double excitations, in contrast to IMOM, leading to a slightly smaller Hamiltonian. For the sake of reproducibility, it should be noted that the assignment of π*­(a_g_) is different from that stated in Table 2 for HF/def2-TZVP (LUMO + 4 for *n* = 2, 3; LUMO + 2 for *n* = 4) and BHandHLYP/def2-TZVP (LUMO + 2 for *n* = 2).

**Table 2:** Excitation Patterns Used for the Generation of Orbital-Optimized States for NOCI Calculations of Polyenes C_2*n*_H_2*n*+2_ ^a^

	requires spin-purification	
determinant	NOCI-IMOM	NOCI-COOX
ground-state KS/HF determinant	no	no
Single Excitations		
π(a_g_) → π*(b_u_)	yes	yes
π(a_g_) → π*(a_g_)	yes	yes
π(b_u_) → π*(b_u_)	yes	yes
π(b_u_) → π*(a_g_)	yes	yes
Double Excitations of Seniority Zero		
$\pi {\left(a_{g}\right)}^{2}\to \pi^{*}{\left(b_{u}\right)}^{2}$	no	no
$\pi {\left(a_{g}\right)}^{2}\to \pi^{*}{\left(a_{g}\right)}^{2}$	no	no
$\pi {\left(b_{u}\right)}^{2}\to \pi^{*}{\left(b_{u}\right)}^{2}$	no	no
Double Excitations of Seniority Two		
$\pi {\left(a_{g}\right)}^{2}\to \pi^{*}\left(b_{u}\right),\pi^{*}\left(a_{g}\right)$	yes	no
$\pi \left(b_{u}\right),\pi \left(a_{g}\right)\to \pi^{*}{\left(b_{u}\right)}^{2}$	yes	no

a Typical assignments: π­(b_u_) ≡ HOMO – 1, π­(a_g_) ≡ HOMO, π*­(b_u_) ≡ LUMO, π*­(a_g_) ≡ LUMO + 1.

For a first test case, we analyze the vertical 1 ^1^B_u_ and 2 ^1^A_g_ excitation energies for the polyenes C_2*n*_H_2*n*+2_ with 2 ≤ *n* ≤ 10, which we illustrate in Figures 1 and 2 alongside the corresponding $\left\langle {Ŝ}^{2}\right\rangle$ values for Hartree–Fock and PBE0 reference states, respectively. We also include ADC(3) results computed using the Q-Chem 5.1 program package^89^ for comparison. All geometries were optimized at the B3LYP-D3­(BJ)/def2-TZVP level. Considering Figures 1 and 2, the lack of double-excitations in the CIS/TDA-TDDFT approach is immediately apparent, as the 2 ^1^A_g_ excitation energy decreases too slowly with respect to chain length *n*, particularly for CIS. NOCI-IMOM and NOCI-COOX perform mostly on par; notably, for HF-based reference states, the 2 ^1^A_g_ state exhibits quite severe spin-contamination for the longer polyenes. As alluded to before, the spin-purification procedures of Sundstrom and Head-Gordon^56^ or Lee and Thom^60^ could be used to ameliorate this issue in exchange for more basis states in the Hamiltonian and thus higher computational cost if HF reference states are desired. In contrast, the DFT-based NOCI-COOX calculations exhibit considerably less spin-contamination even for larger *n,* as illustrated for the PBE0 results in Figure 2 (the same applies for calculations based on PBE and BHandHLYP, see Figures S1 and S2 in the Supporting Information). This result is to be expected since Hartree–Fock and hybrid functionals with large admixture of EXX are known to be more prone to spin-contamination;^90^ indeed, our HF reference states themselves are considerably more spin-contaminated with $\left\langle {Ŝ}^{2}\right\rangle >2$ for *n* = 10 compared to PBE, PBE0, and, to a lesser degree, BHandHLYP.

**Figure 1:**
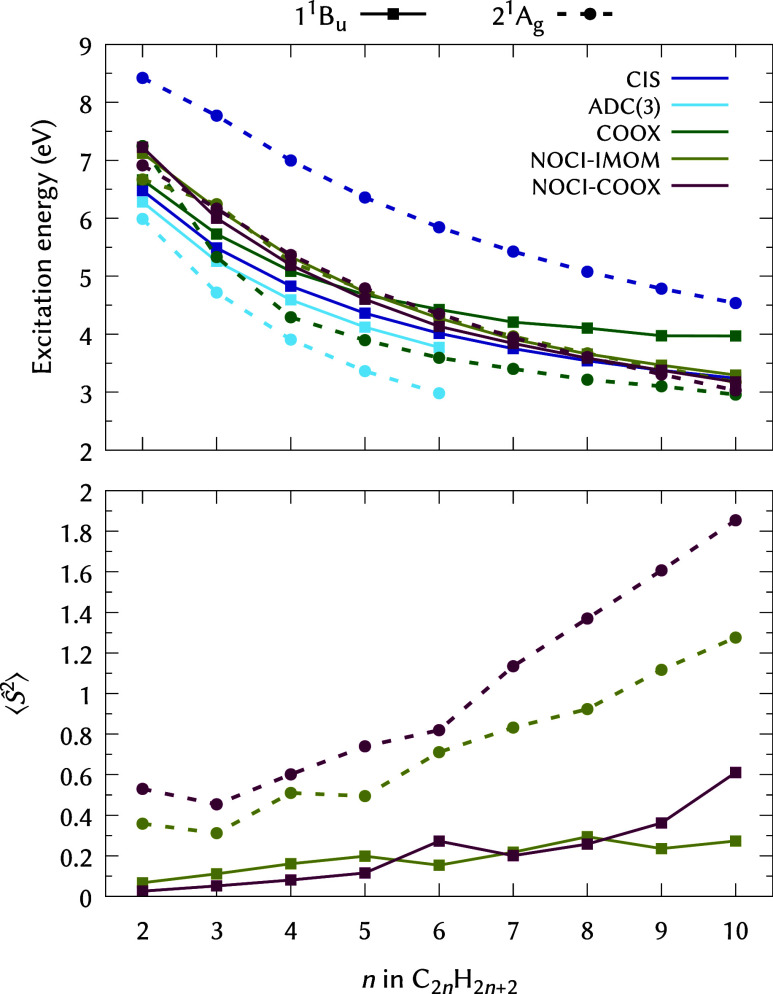
Vertical excitation energies and $\left\langle {Ŝ}^{2}\right\rangle$ values for the 1 ^1^B_u_ (full lines) and 2 ^1^A_g_ (dashed lines) states of linear polyenes C_2*n*_H_2*n*+2_ computed using ADC(3) and various methods at the HF/def2-TZVP level (NOCI-IMOM/NOCI-COOX: 16/14 configurations, see Table 2). Note that CIS, ADC(3), and plain COOX calculations are spin-restricted and thus all satisfy $\left\langle {Ŝ}^{2}\right\rangle =0$ .

**Figure 2:**
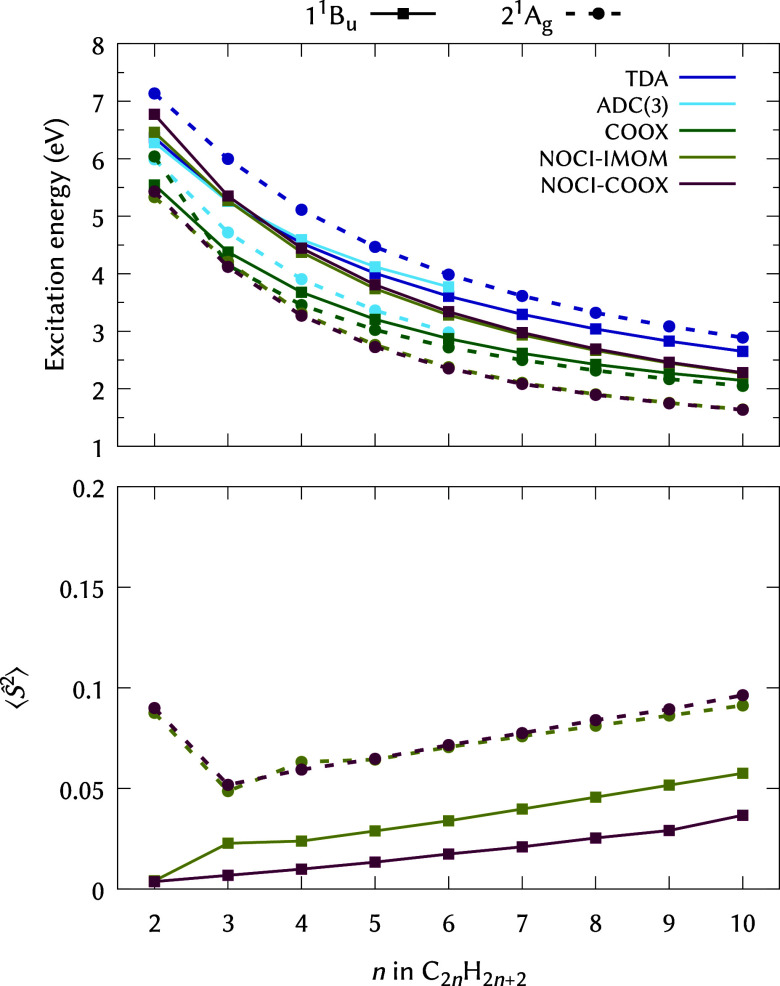
Vertical excitation energies and $\left\langle {Ŝ}^{2}\right\rangle$ values for the 1 ^1^B_u_ (full lines) and 2 ^1^A_g_ (dashed lines) states of linear polyenes C_2*n*_H_2*n*+2_ computed using ADC(3) and various methods at the PBE0/def2-TZVP level (NOCI-IMOM/NOCI-COOX: 16/14 configurations, see Table 2). Note that TDA, ADC(3), and plain COOX calculations are spin-restricted and thus all satisfy $\left\langle {Ŝ}^{2}\right\rangle =0$ .

It should also be highlighted that the plain COOX approach with scaled constraints and Fermi smearing performs remarkably well for these test cases and yields qualitatively correct curves for the vertical excitation energies for HF. For PBE0, the 2 ^1^A_g_ energy also correctly falls below the 1 ^1^B_u_ state, although the gap is comparatively small. Naturally, the ground-state energy remains entirely unaffected.

The computational cost corresponding to Figure 1 is shown in Figure 3. Evidently, NOCI-COOX represents a much more affordable ansatz than ADC(3), while being only about 1 order of magnitude more expensive than CIS. The greater algorithmic stability of ΔCOOX compared to ΔSCF-type approaches is reflected in the much larger SCF timings for NOCI-IMOM, corresponding to a larger number of required iterations to achieve convergence. Combined with the slightly lower number of determinants in the NOCI-COOX Hamiltonian, this results in NOCI-COOX being less expensive than NOCI-IMOM by roughly a factor of 2. It should be kept in mind, however, that the computational cost for NOCI-COOX and NOCI-IMOM heavily depends on the number of determinants included in the Hamiltonian, which in this case is comparatively small, so that the underlying SCF calculations actually constitute the majority of the overall computation time.

**Figure 3:**
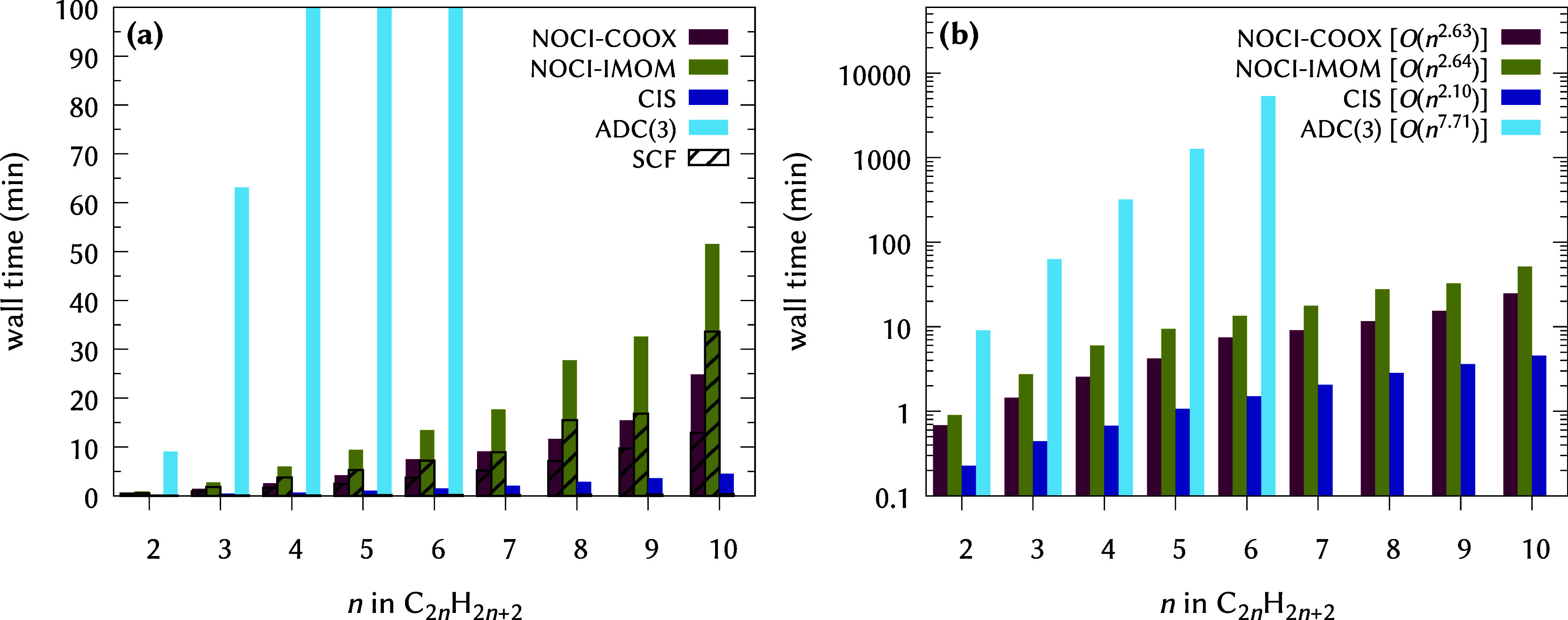
Computational cost for all-*E*-polyenes for different methods based on HF/def2-TZVP reference states on (a) linear and (b) logarithmic scales. In (a), the timings for the necessary SCF calculations are highlighted. In (b), the key includes the approximate scaling behavior with respect to the chain length *n*. For ADC(3), two roots were computed; for CIS, five roots in each of the two irreducible representations (B_u_ and A_g_) were computed; for NOCI-IMOM/NOCI-COOX, the 16/14 reference states according to Table 2 were included.

Of course, the rather small manual excitation space used for Figures 1–3 does not represent an ideal choice, particularly for increasing chain lengths. Therefore, we investigated an automatic selection of the excitation space for the reference geometry of butadiene based on an orbital energy difference criterion as defined in eq 30, which we illustrate in Figure 4 for PBE0 reference states, where we also include an “orthogonal CI” ansatz (OCI) using orthogonal Slater determinants |Φ_*i...*_^*a...*^⟩ in place of orbital-optimized states with the same approximate Hamiltonian as in NOCI (this may be interpreted as a kind of selected CI with singles and doubles). Both NOCI-COOX and OCI feature a considerable lowering of the 2 ^1^A_g_ energy between thresholds of 11 and 12 eV, coinciding with the inclusion of the important HOMO^2^ → LUMO^2^ double excitation in the configuration space, and a second, smaller drop between 16 and 17 eV that corresponds to the inclusion of the (HOMO – 1)^2^ → LUMO^2^ double excitation. The effect of variational relaxation by virtue of COOX is clearly visible from the lower absolute energies of NOCI-COOX compared to OCI. The 1 ^1^B_u_ and 2 ^1^A_g_ excitation energies appear to be converged to a reasonable degree of accuracy for Δε_max_ = 20 eV. For a good compromise between accuracy and efficiency, a slightly narrower window, e.g., Δε_max_ = 18 eV, may be chosen due to the growing size of the effective Hamiltonian—in the sampled range from 10 to 20 eV, the number of NOCI states grows approximately as O­(Δε_max_^4^) as shown in Figure 4c. This is directly reflected in the resulting computation time: Recall that the construction of the NOCI Hamiltonian scales with the square of the number of determinants included; therefore, the overall scaling with respect to Δε_max_ should be expected to be approximately *O*(Δε_max_^8^), which matches well with the actual computation time shown in Figure 5. For Δε_max_ = 18 eV, the excitation energies deviate by less than 0.1 eV from those for Δε_max_ = 20 eV and less than 0.2 eV from those for Δε_max_ = 25 eV, but they require less than half the computation time of Δε_max_ = 20 eV and less than one-tenth of Δε_max_ = 25 eV. Therefore, the choice of an adequate value for Δε_max_ is a critical factor to achieve calculations that are both accurate and computationally viable. It should further be noted that for very large values of Δε_max_, numerical instabilities in the NOCI Hamiltonian can arise due to SVD. In these cases, a looser orthogonality threshold for the singular values should be used.

**Figure 4:**
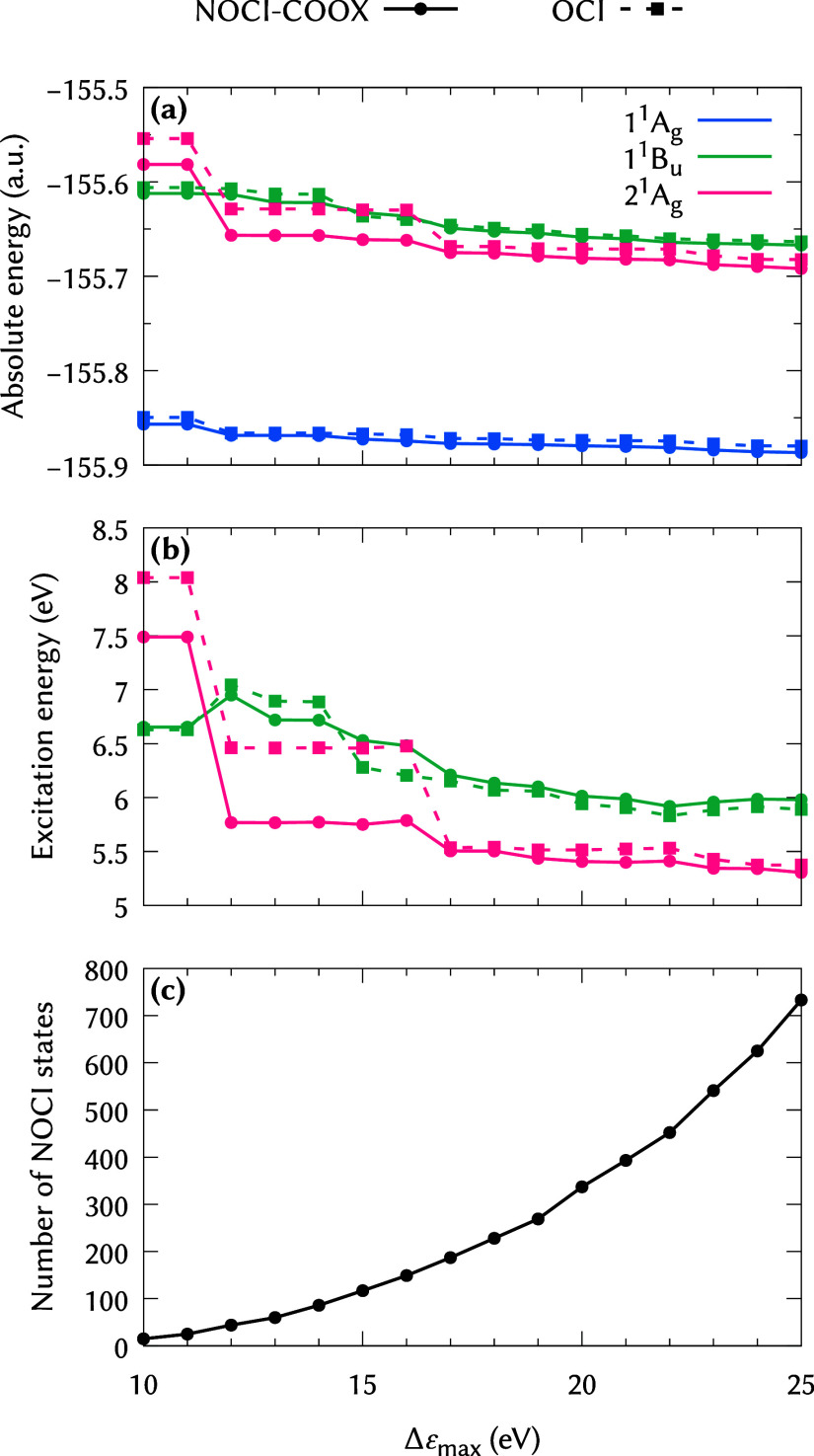
(a) 1 ^1^A_g_, 1 ^1^B_u_, and 2 ^1^A_g_ energies, (b) 1 ^1^B_u_ and 2 ^1^A_g_ excitation energies, and (c) number of NOCI states for butadiene with PBE0/def2-TZVP reference states for different selection criteria Δε_max_. OCI denotes an orthogonal CI ansatz using the same approximate Hamiltonian as for NOCI-COOX (see text for further details).

**Figure 5:**
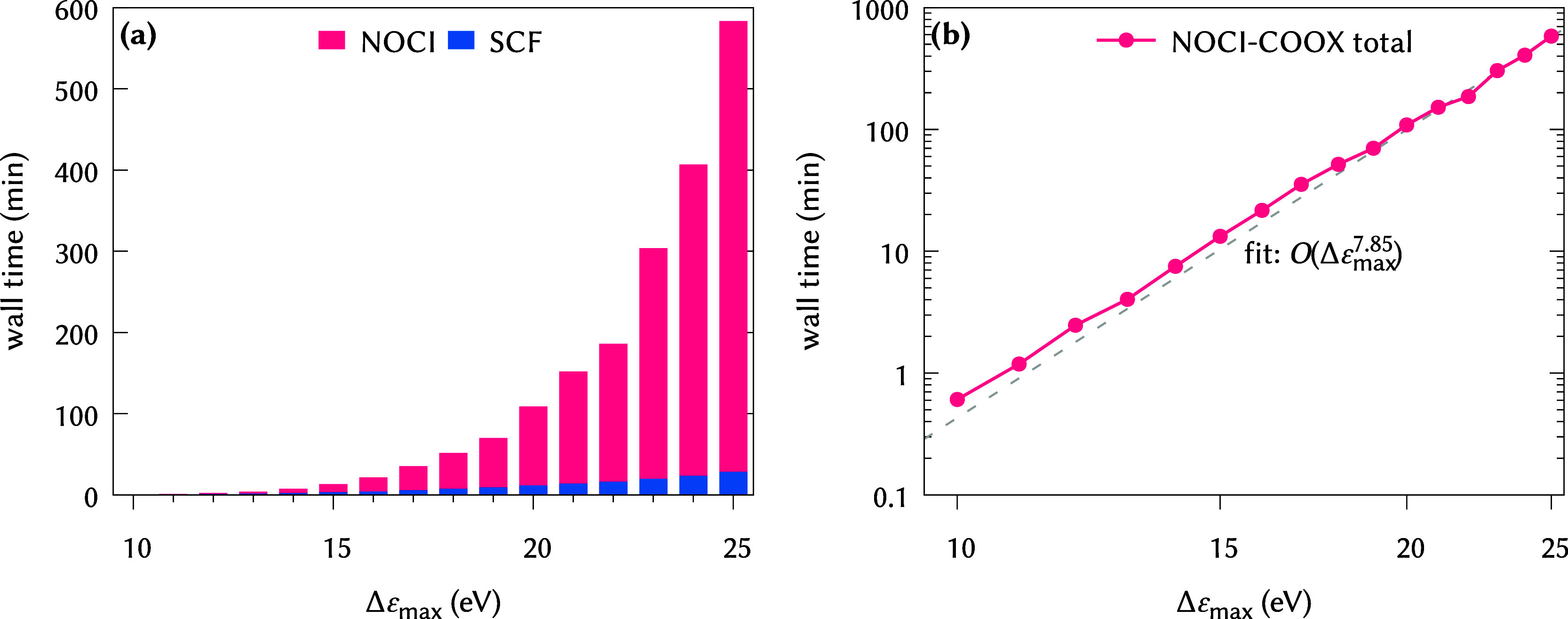
Computational cost of NOCI-COOX/PBE0/def2-TZVP for butadiene as a function of the selection criterion Δε_max_. (a) Linear scale with partitioning into SCF and NOCI parts and (b) logarithmic scale for the total time.

We stress that the timings shown in Figures 3 and 5 represent timings on a single compute node. As the optimization of ΔCOOX reference states and computation of matrix elements $H_{IJ}$ have no internal data dependencies, the distribution across multiple compute nodes should be a straightforward way to further lower the computational cost of NOCI-COOX.

Figure 6 shows the energy profiles for the aforementioned BLA mode of butadiene, which consists of a stretching of the double bonds and simultaneous compression of the single bond, for Hartree–Fock reference states evaluated with the def2-TZVPD basis set^72,91^ (for other functionals, see Figures S3–S5 in the Supporting Information). State-averaged CASPT2 calculations (Figure 6a) of Levine et al.^17^ have shown that the 2 ^1^A_g_ state features a minimum at considerably higher BLA than the 1 ^1^B_u_ and 3 ^1^A_g_ states, which CIS (Figure 6c) clearly fails to reproduce, once again highlighting the insufficiency of the linear-response ansatz in cases of significant double-excitation character. Likewise, the scaled COOX approach (Figure 6d) fails to predict the correct behavior for the 2 ^1^A_g_ state and exhibits some fluctuations along the BLA coordinate. Furthermore, for BHandHLYP/def2-TZVP, an instability in the energetically higher 3 ^1^A_g_ state is observed because the excited-state character changes along the alternation coordinate (see Figures S6 and S7 in the Supporting Information). Surprisingly, even ADC(3) (Figure 6b) struggles for this system, exhibiting unphysical bumps for all states at an alternation of 0.105 Å and deviating strongly from the CASPT2 curves for the 2 ^1^A_g_ and 3 ^1^A_g_ states, with the former almost monotonically decreasing in energy and the latter being energetically too close to the 1 ^1^B_u_ state.

**Figure 6:**
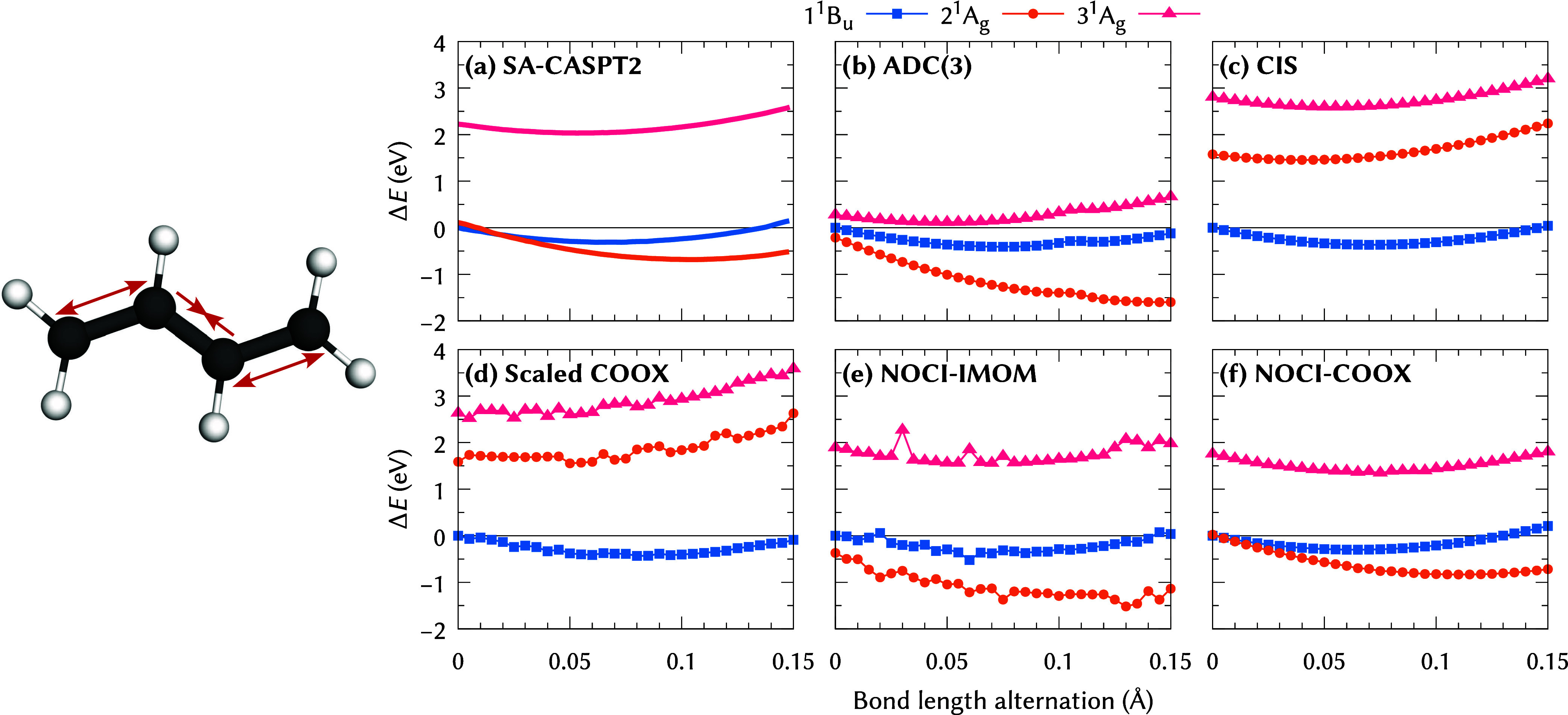
BLA of butadiene. (a) State-averaged CAS­(4,4)-PT2 data (6-31G** basis set) adapted from ref 17; (b) computed at the ADC(3)/def2-TZVPD level; (c–f) computed using HF/def2-TZVPD reference states (NOCI-IMOM/NOCI-COOX: 16/14 configurations, see Table 2). Energies relative to the 1 ^1^B_u_ energy at the reference geometry.

For the NOCI approaches, the IMOM-based results (Figure 6e) suggest a qualitatively correct behavior but clearly exhibit major numerical instabilities for the crucial 1 ^1^B_u_ and 2 ^1^A_g_ states, which preclude any quantitative analysis. It is interesting to note that these discontinuities seem to arise from numerical instabilities in the NOCI eigenvalue problem rather than the orbital-optimized states themselves, which produce mostly smooth curves as shown in Figure S8 in the Supporting Information. In contrast, the NOCI-COOX calculations (Figure 6f) yield physically correct and smooth curves along the full BLA coordinate, again underlining the suitability of constraint-based orbital-optimized reference states as opposed to ΔSCF-type approaches for nonorthogonal configuration interaction.

Finally, we briefly touch upon the functional dependence of our NOCI-COOX approach. To that extent, we computed the 1 ^1^B_u_, 2 ^1^A_g_, 1 ^3^B_u_, and 1 ^3^A_g_ excitation energies for butadiene, *E*-hexatriene, and all-*E*-octatetraene for a variety of functionals from separate rungs of Jacob’s ladder. To gauge the effect of higher EXX admixture, we compare several functionals based on the generalized gradient approximation PBE functional,^92^ namely, PBE itself (no EXX), PBE0 (25% global EXX), PBE50 (50% global EXX),^93^ LRC-ωPBE (0% short-range EXX, 100% long-range EXX, ω = 0.3*a*_0_^–1^),^94^ and LRC-ωPBEh (20% short-range EXX, 100% long-range EXX, ω = 0.2*a*_0_^–1^).^94^ Furthermore, we include the SVWN5 functional^95,96^ as a representative of local density approximations, BHandHLYP (50% global EXX), ωB97X (15.771% short-range EXX, 100% long-range EXX, ω = 0.3*a*_0_^–1^),^97^ and HF. Table 3 shows the mean errors and mean absolute errors relative to the theoretical best estimates (TBE) of Thiel and co-workers,^98,99^ where we have used the reference excitation space defined in Table 2 and the def2-TZVPD basis set in all NOCI calculations. Note that for better comparison to the TBE and DFT/MRCI values, we used the geometries from ref 98 rather than our optimized geometries used above.

**Table 3:** Mean Errors (ME) and Mean Absolute Errors (MAE) in eV for the Lowest-Lying Singlet and Triplet States of Butadiene, *E*-Hexatriene, and All-*E*-Octatetraene Compared to the TBE from ref 99

	ME					MAE				
method	1 ^1^B_u_	2 ^1^A_g_	1 ^3^B_u_	1 ^3^A_g_	total	1 ^1^B_u_	2 ^1^A_g_	1 ^3^B_u_	1 ^3^A_g_	total
Reference										
CASPT2^a^	–0.01	+0.06	+0.15	+0.04	+0.06	0.20	0.17	0.15	0.04	0.14
DFT/MRCI^b^	–0.24	–0.33	–0.07	–0.22	–0.22	0.24	0.33	0.11	0.22	0.22
ADC(3)^c^	+0.01	–0.63	–0.11	–0.15	–0.22	0.08	0.63	0.11	0.15	0.24
NOCI-COOX^d^										
SVWN5	–0.04	–1.73	–1.40	–1.69	–1.22	0.28	1.73	1.40	1.69	1.28
PBE	–0.06	–1.79	–1.40	–1.74	–1.25	0.28	1.79	1.40	1.74	1.30
PBE0	+0.13	–1.15	–0.85	–1.12	–0.75	0.30	1.15	0.85	1.12	0.85
PBE50	+0.34	–0.46	–0.29	–0.45	–0.22	0.35	0.46	0.29	0.45	0.39
LRC-ωPBE	+0.51	–0.37	–0.26	–0.42	–0.13	0.51	0.37	0.26	0.42	0.39
LRC-ωPBEh	+0.39	–0.58	–0.41	–0.62	–0.30	0.39	0.58	0.41	0.62	0.50
BHandHLYP	+0.31	–0.45	–0.28	–0.42	–0.21	0.33	0.45	0.28	0.42	0.37
ωB97X	+0.60	–0.12	–0.05	–0.17	+0.06	0.60	0.17	0.11	0.22	0.27
HF	+0.65	+0.80	+0.88	+0.98	+0.83	0.65	0.80	0.88	0.98	0.83
NOCI-IMOM^d^										
SVWN5	–0.35	–1.67	–1.45	–1.81	–1.32	0.35	1.67	1.45	1.81	1.32
PBE	–0.36	–1.77	–1.47	–1.87	–1.37	0.36	1.77	1.47	1.87	1.37
PBE0	–0.02	–1.16	–1.06	–1.21	–0.86	0.20	1.16	1.06	1.21	0.91
PBE50					n.c.^e^					
LRC-ωPBE					n.c.^e^					
LRC-ωPBEh					n.c.^e^					
BHandHLYP					n.c.^e^					
ωB97X					n.c.^e^					
HF					n.c.^e^					

a aug-cc-pVTZ basis set, data from ref 99.

b Ahlrichs TZVP basis set, data from ref 98.

c def2-TZVP basis set, this work.

d def2-TZVPD basis set, reference states as defined in Table 2.

e IMOM calculations did not converge within 500 iterations.

As one might expect, the results in Table 3 show a clear trend toward higher excitation energies with increasing admixture of EXX. The semilocal SVWN5 and PBE functionals considerably underestimate the excitation energies by more than 1 eV on average, whereas PBE0 yields overall better results but still systematically underestimates the excitation energies. NOCI-COOX calculations based on range-separated hybrids, in particular, LRC-ωPBE and ωB97X, perform very well, providing ME and MAE values that are mostly on par with the much more computationally involved CASPT2, DFT/MRCI, and ADC(3) methods. For NOCI-IMOM, we once again face significant numerical instabilities for all hybrids except PBE0, which precludes a meaningful calculation of ME and MAE values. The individual energies for the systems where convergence could be achieved are listed in Table S1 of the Supporting Information for comparison. For SVWN5, PBE, and PBE0, NOCI-IMOM performs slightly worse than NOCI-COOX on average, generally yielding slightly lower excitation energies, likely due to the different treatment of doubly excited determinants of seniority two.

While these results show clear trends, they are of course not to be mistaken for a comprehensive benchmark, which would need to cover a far greater number of variables (more systems, larger reference spaces, dependence on basis sets, dependence on Δε_max_,...) and is therefore far beyond the scope of this work. Nevertheless, comprehensive benchmarking should be undertaken in future work to better understand the strengths and weaknesses of the NOCI-COOX approach as opposed to those of ΔSCF-based NOCI approaches, LR-TDDFT, and higher-level methods. Furthermore, the optimal tuning of range-separation parameters is a widely used trick in applications of ΔSCF-type methods and could represent an intriguing avenue to further improve the accuracy of NOCI-COOX.

### 4.2 Vanishing Reference States: Lithium Fluoride Dissociation

A major problem in ΔSCF-based NOCI calculations is the potential disappearance of reference states, which can lead to discontinuities on the potential energy surface. For example, Thom and Head-Gordon^55^ observed that the two lowest-lying orbital-optimized Σ^+^ states of lithium fluoride, obtained through an SCF metadynamics approach,^100^ coalesce and vanish at an interatomic separation of 3.15 Å. This phenomenon can be seen in Figure 7, where we have computed the LiF dissociation profile at the HF/def2-TZVPPD^72,91^ level using NOCI-IMOM and NOCI-COOX, respectively. Besides some general numerical instabilities, the IMOM state obtained from the σ → σ* excitation does indeed vanish for *R*_Li–F_ < 3.15 Å, resulting in jump discontinuities in the 1 ^1^Σ^+^ and 2 ^1^Σ^+^ states upon diagonalization of the NOCI Hamiltonian. It is also noteworthy how much the σ → σ* IMOM state deviates from $\left\langle {Ŝ}^{2}\right\rangle =1$ , with values of $\left\langle {Ŝ}^{2}\right\rangle =0.6538$ at *R*_Li–F_ = 3.10 Å and $\left\langle {Ŝ}^{2}\right\rangle =0.8493$ at *R*_Li–F_ = 3.15 Å, respectively. In contrast, there are no disappearing states in the corresponding ΔCOOX calculations; thus, we obtain a smooth dissociation profile which, like NOCI-IMOM, features the expected avoided crossing but does not contain the problematic jump discontinuities at smaller interatomic separation. We assume that this distinction originates from the fact that ΔCOOX, in contrast to ΔSCF-based approaches, does not require the excited-state density to be a stationary solution of the unperturbed Fock matrix. Therefore, we can obtain real-valued reference states through the regular ΔCOOX machinery without the need to invoke analytic continuation in the vein of holomorphic Hartree–Fock theory.^101,102^

**Figure 7:**
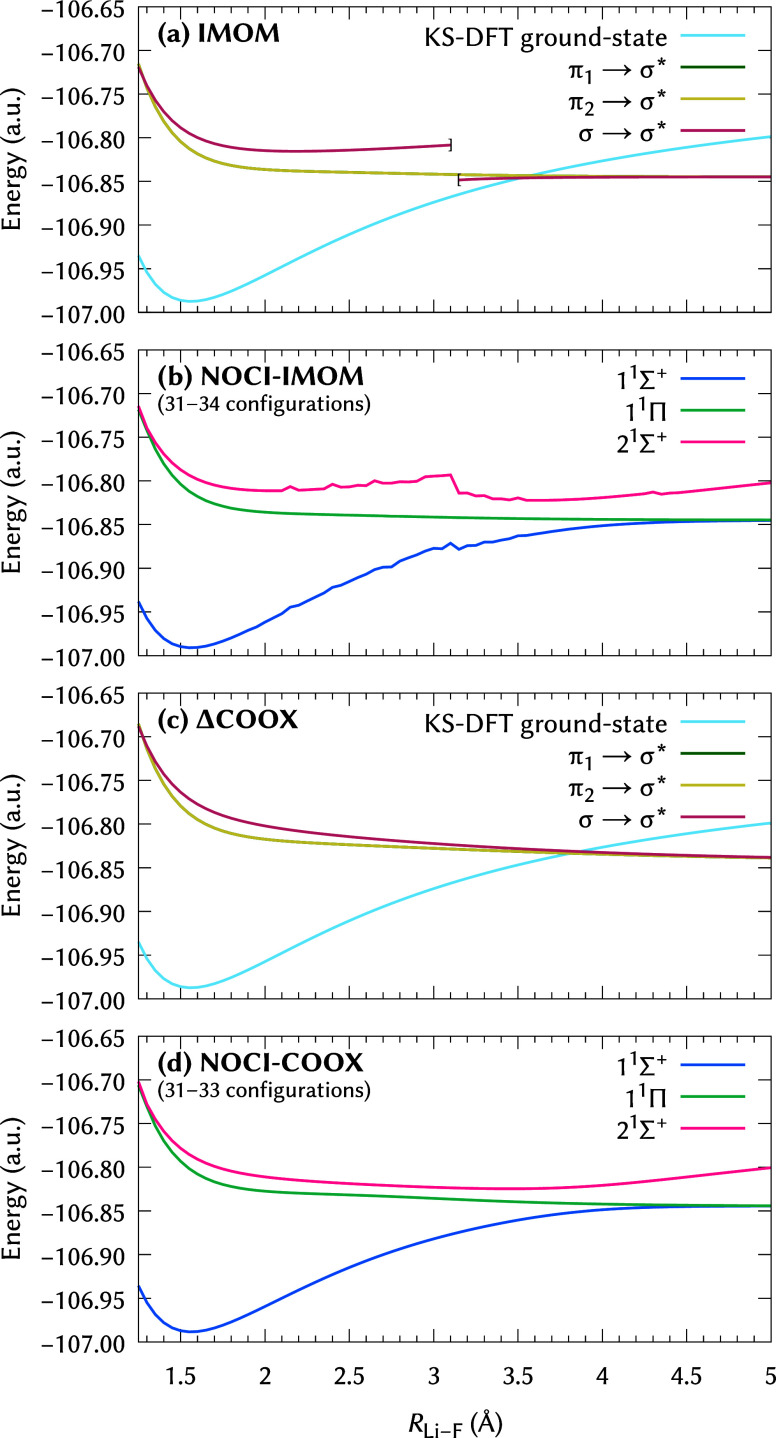
Dissociation profile of LiF computed at the HF/def2-TZVPPD level of theory. Reference states were selected using Δε_max_ = max­{2­(ε_LUMO_ – ε_HOMO_), 10 eV} between HOMO – 2 and LUMO + 4. The four energetically lowest IMOM and ΔCOOX states included in the NOCI Hamiltonian are shown in (a,c), respectively. The resulting three lowest NOCI states are shown in (b,d), respectively. Note that the orbital-optimized π_1_ → σ* and π_2_ → σ* states are degenerate for both IMOM and ΔCOOX.

### 4.3 Conical Intersections: Ethylene Pyramidalization and Azobenzene Isomerization

Conical intersections (CoIns) represent one of the most important mechanisms for ultrafast nonradiative relaxation from electronically excited states. For instance, the presence of CoIns near the Franck–Condon point plays a crucial role in the photostability of the nucleobases, which form the building blocks of DNA and RNA.^103–106^ As such, the study of CoIns by quantum-chemical means is indispensable but poses some obvious challenges: Besides the breakdown of the Born–Oppenheimer approximation at near-degeneracies, wave functions in the vicinity of conical intersections are inherently multiconfigurational, which precludes the use of linear-response approaches for S_0_/S_1_ CoIns since the ground-state remains entirely uncorrelated as a direct consequence of Brillouin’s theorem, whereas popular multireference methods such as CASSCF and CASPT2, besides their comparatively high computational cost, require manual fine-tuning to ensure correct active space definitions.

We present NOCI-COOX results for two commonly studied systems, i.e., the pyramidalization of twisted ethylene and the cis–trans-isomerization of azobenzene) to investigate whether NOCI may provide a valuable alternative to the aforementioned methods.

The pyramidalization from the twisted *D*_2*d*_ geometry of ethylene has been understood for decades,^107,108^ and the failure of LR-TDDFT to accurately reproduce the conical intersection along the pyramidalization coordinate is well-documented.^17^ Recent modifications of the TDA-TDDFT Hamiltonian to incorporate correlation with the ground-state have been shown to rectify this behavior,^109,110^ but of course still fall short in cases where double-excitation character plays a role. In Figure 8, we show the S_0_ and S_1_ energies obtained with NOCI-COOX from a variety of reference methods. As noted by other authors,^109,110^ KS-DFT exhibits instabilities in the transition from the *D*_2*d*_ geometry (θ ≈ 30°), which arise from RHF/RHF-type wave function instabilities in the restricted ground-state determinant at intermediate angles. While RHF/UHF-type instabilities are accounted for by virtue of the NOCI-COOX approach, the RHF/RHF instabilities produce discontinuous NOCI energy profiles as shown in Figure S9 in the Supporting Information. To rectify these instabilities, we perform a reverse scan starting at θ = 130°, where the KS-DFT ground-state does not exhibit RHF/RHF instabilities, and always use the KS-DFT density from the previous geometry as the initial guess.

**Figure 8:**
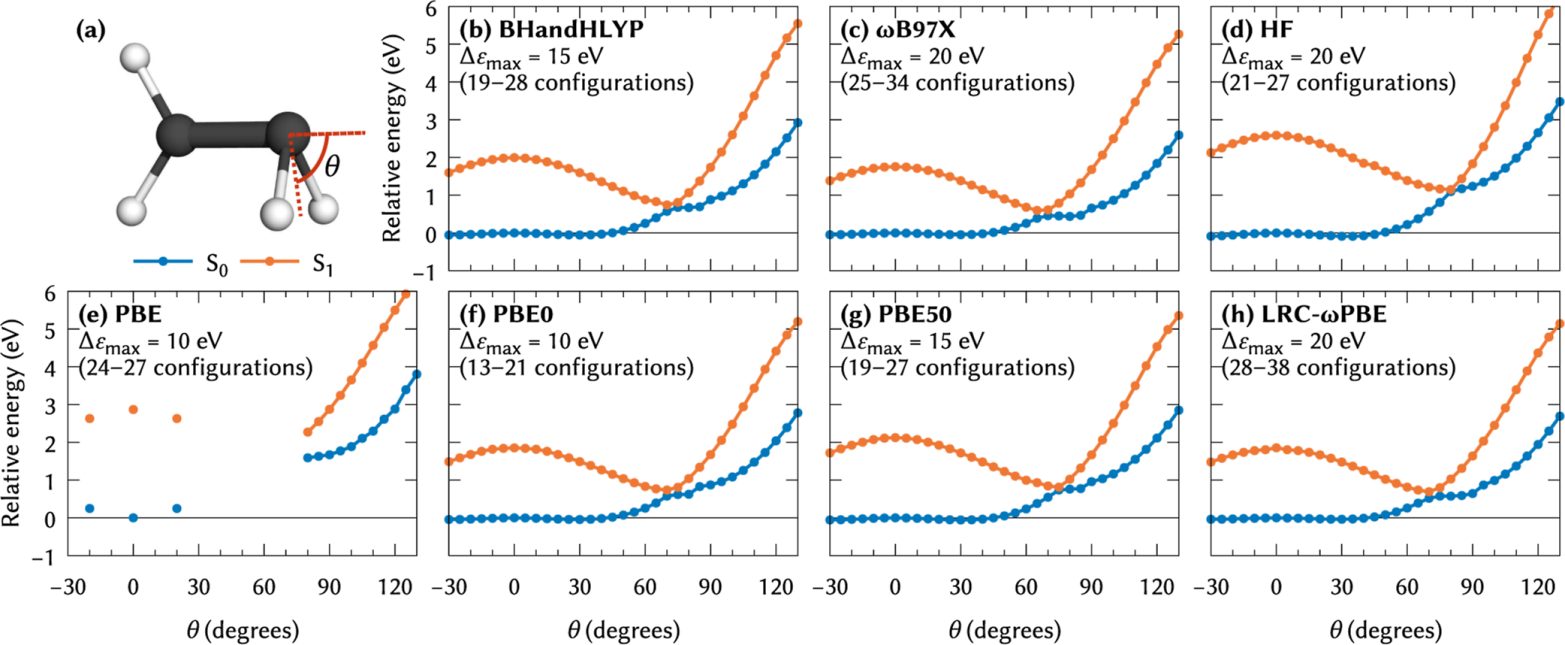
Relative S_0_ and S_1_ energies along the pyramidalization coordinate of twisted ethylene shown in (a), computed using NOCI-COOX with the def2-TZVP basis set and (b) BHandHLYP, (c) ωB97X, (d) HF, (e) PBE, (f) PBE0, (g) PBE50, and (h) LRC-ωPBE reference states. The active orbital space has been restricted from HOMO – 3 through LUMO + 3.

With the exception of PBE, where the KS ground-state routinely fails to converge, all methods shown in Figure 8 correctly yield vanishing energy gaps between S_0_ and S_1_ at pyramidalization angles of 70° to 80° relative to the *D*_2*d*_ geometry. The HF-based curves in particular show very good agreement with the MRCI results from ref 110. For the DFT-based results, we obtain mostly similar results, with PBE50 and BHandHLYP generally yielding slightly larger gaps than the other functionals.

Next, we discuss the S_0_–S_1_ CoIn encountered during the cis–trans-isomerization of azobenzene, which, due to its high photopharmaceutical promise, has been the subject of numerous quantum-chemical studies^111–117^ and is routinely used as a test system for excited-state methods.^51,110,113,118^ The isomerization, which is achieved through a combination of torsion of the central CNNC dihedral and in-plane inversion of one of the CNN angles, again poses considerable challenges for linear-response methods. While these can be ameliorated to some degree through the use of Fermi-smearing TDA,^118^ alternative approaches in the form of orbital-optimized methods have proven to be promising.^51,113^ In a previous work,^51^ we have established that, in contrast to other cDFT approaches, COOX retains correct S_0_/S_1_ state ordering along the CNNC torsion and yields physically sound excited-state densities which can be used, e.g., for the computation of molecular gradients or the application of post-SCF correlation. However, the COOX approach does, of course, not in any form include correlation with the ground-state; therefore, a reassessment using NOCI-COOX is warranted. To sample both the torsion and inversion coordinate, we used a series of relaxed scans at the B3LYP-D3­(BJ)/def2-TZVP level, in which one CNN angle was fixed at 120° and the CNNC and NNC angles were varied (see Figure 9a for a graphical representation). For each geometry, we then evaluated NOCI-COOX energies using BHandHLYP reference states with a selection threshold of Δε_max_ = 15 eV and a reduced active orbital space from HOMO – 2 through LUMO + 2. Figure 9b shows the resulting potential energy surfaces for S_0_ and S_1_, with the corresponding excitation energies displayed in Figure 9c. The smallest energy gap is obtained at ∠NNC = 140°, ∠CNNC = 92°, as highlighted in Figure 9d, which is in good agreement with estimates from static CASSCF calculations^112^ and nonadiabatic molecular dynamics simulations.^119^

**Figure 9:**
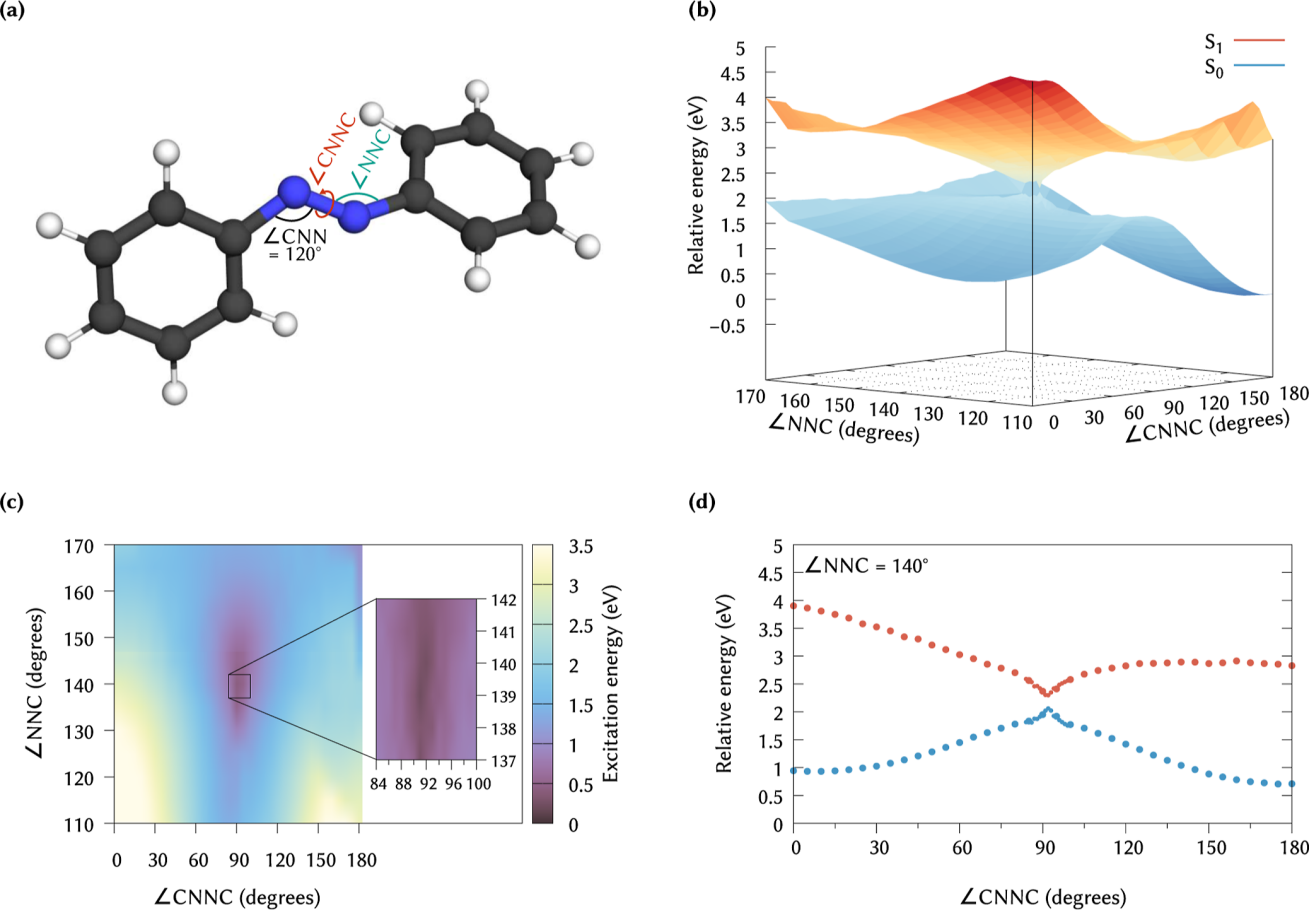
(a) Schematic representation of the scan coordinates; ∠CNN is fixed at 120°. (b) Potential energy surfaces of the S_0_ and S_1_ states for different angles ∠NNC and dihedrals ∠CNNC, energies relative to the S_0_ at ∠NNC = 120°, ∠CNNC = 180°. (c) S_1_ excitation energies (S_1_–S_0_), and (d) S_0_ and S_1_ energies for fixed ∠NNC = 140°. Data generated using NOCI-COOX/BHandHLYP/def2-TZVP with Δε_max_ = 15 eV and a reduced orbital space from HOMO – 2 through LUMO + 2 (23–35 ΔCOOX configurations).

### 4.4 Treatment of Complex Environments

The NOCI-COOX approach can easily be adapted to our recently proposed constraint embedding scheme, termed “e-COOX”,^53^ to enable the targeted computation of excited states in complex environments. In e-COOX, the constraint potentials are computed in a predefined subsystem and subsequently embedded into the basis of the complete system to ensure orthogonality of the relevant orbital spaces

31 $$\forall i,a:\varphi_{i}^{sub}⊥\varphi_{a}^{full},,\varphi_{a}^{sub}⊥\varphi_{i}^{full},,\varphi_{i}^{sub}⊥\varphi_{a}^{sub}$$

followed by the variational COOX calculation in the full quantum system. To illustrate the application of the e-COOX scheme, we computed the solvatochromic shifts of the lowest *n* → π* and π → π* excitations of uracil upon solution in water, using geometries with 25 and 100 explicit water molecules, which were constructed in ref 53 using the quantum cluster growth algorithm^120^ of the crest program package.^121,122^ Furthermore, we compare the results to the widely used conductor-like polarizable continuum model^123^ for implicit solvation; the corresponding vacuum and PCM geometries were optimized at the B2PLYP-D3­(BJ)^124^/def2-TZVP level of theory. For the PCM calculations, we employ the perturbative state-specific (ptSS-PCM) and linear-response (ptLR-PCM) approaches of Mewes et al.^125^ for the description of nonequilibrium solvent effects upon vertical excitation. In the case of NOCI-COOX, we use a “perturbation-density” scheme,^125^ i.e., only the ground-state KS-DFT calculation is carried out with PCM enabled, the NOCI Hamiltonian is then constructed using the resulting polarized orbitals, and the ptSS/ptLR energies are computed from the respective NOCI-COOX ground-state, excited-state, and transition densities, as defined through eq 15 following diagonalization. Calculations were performed with the PBE0 functional and the def2-SVP basis set with an energy selection criterion of Δε_max_ = 14 eV. For the isolated uracil subsystem in the e-COOX approach, this results in 58 single excitations, 4 double excitations of seniority zero, and 7 double excitations of seniority two, which need to be variationally optimized and included in the NOCI Hamiltonian, yielding a total of 128 ΔCOOX configurations with little sensitivity to the size of the solvation shell. In contrast, if we were to consider the full quantum system including all 100 water molecules using the same energy threshold, we would obtain 9939 single excitations, 38 double excitations of seniority zero, and 1493 double excitations of seniority two for a total of 21,410 ΔCOOX configurations, leading to a dramatic increase of computational cost in the evaluation of the NOCI Hamiltonian as well as severe difficulty to identify the excited states of interest, while providing little to no gain in accuracy for the relevant *n* → π* and π → π* states. Likewise, the system with 25 water molecules would require the inclusion of 2492 ΔCOOX configurations, far outweighing the corresponding 131 configurations needed in the e-COOX approach.

We list the excitation energies and solvatochromic shifts in Table 4 alongside CC2 results from Olsen et al.^126^ and EOM-CCSDt­(II) results from Epifanovsky et al.^127^—a comparison to NOCI-IMOM was not possible due to convergence problems for a large number of higher-lying IMOM states. In the vacuum, the application of NOCI on top of the plain COOX scheme evidently yields far better agreement with CC2 and the TBE of Thiel and co-workers.^99^ Regarding the solvatochromic shifts, NOCI-COOX—like regular e-COOX—yields comparatively high shifts for the dark *n* → π* state when using the e-COOX embedding scheme, though the total excitation energy more closely matches the higher-level reference methods. IMOM and ΔCOOX yield results comparable to each other, with the former showing better agreement for the vacuum π → π* energy and the latter predicting the vacuum *n* → π* state more accurately, while the computed solvatochromic shifts are very similar. In fact, all PCM-based methods, with the exception of the regular closed-shell COOX approach, give a shift of around −0.2 eV for the π → π* state in excellent agreement with CC2, but incorrectly predict an even larger red-shift for the *n* → π* state. It should be noted that for the bright π → π* state, the equation-of-motion coupled-cluster calculations from ref 127 have been reported to yield a slight blue-shift of +0.07 eV,^127^ which we also observe in NOCI-COOX with the larger 100 molecule solvation shell. Since the sign of the NOCI shift changes going from 25 to 100 explicit water molecules, and since a majority of theoretical and experimental values agree on small red-shifts for the π → π* state (see, e.g., ref 126 and references therein), a more in-depth analysis using several snapshots from molecular dynamics simulations would be needed to reach a definitive conclusion, as deviations of the uracil geometry in our single crest-optimized structures may lead to biased excitation energies. However, such an in-depth study is clearly beyond the scope of this work.

**Table 4:** Vertical Excitation Energies and Solvatochromic Shifts (in eV) of the *n* → π* and π → π* Excitations of Uracil in Water, Computed Using PBE0/def2-SVP Reference States^a^

method	environment	*n* → π*	π → π*
IMOM	vacuum	4.53	5.17
	water (ptSS-PCM)	4.12 (−0.41)	4.97 (−0.20)
ΔCOOX	vacuum	4.77	5.02
	water (ptSS-PCM)	4.29 (−0.48)	4.79 (−0.23)
COOX	vacuum	5.66	6.32
	water (e-COOX@25 H_2_O)	6.61 (+0.95)	6.44 (+0.12)
	water (e-COOX@100 H_2_O)	6.50 (+0.84)	6.70 (+0.38)
	water (ptSS-PCM)	5.35 (−0.31)	5.70 (−0.62)
NOCI-COOX	vacuum	4.64	5.30
	water (e-COOX@25 H_2_O)	5.80 (+1.16)	5.16 (−0.14)
	water (e-COOX@100 H_2_O)	5.58 (+0.94)	5.40 (+0.10)
	water (ptLR-PCM)	4.42 (−0.22)	5.10 (−0.20)
	water (ptSS-PCM)	4.32 (−0.32)	5.08 (−0.22)
CC2^b^	vacuum	4.93	5.40
	water (QM/MM)	5.37 (+0.43)	5.20 (−0.20)
EOM-CCSDt(II)^c^	vacuum	5.31	5.89
	water (QM/MM)	5.75 (+0.44)	5.96 (+0.07)
best est.^d^	vacuum	5.00	5.25

a Values in parentheses indicate the solvatochromic shift.

b CC2/aug-cc-pVDZ with QM/MM embedding (QM: uracil, MM: water), data from ref 126.

c EOM-CCSDt­(II)/6-31G­(d) with QM/MM embedding (QM: uracil, MM: water), data from ref 127.

d Theoretical best estimates from ref 99.

### 4.5 Core Excitations

As a final application example, we present the near-edge X-ray absorption spectra of formaldehyde computed through NOCI-COOX. Since COOX has already been shown to deliver excellent accuracy for core-excitations,^52^ the NOCI Hamiltonian serves mostly for the purposes of spin-purification in this case: Whereas in regular COOX, we would have to compute both spin-contaminated singlet excitations and their corresponding triplet states to apply an approximate spin-projection formula, using NOCI, we can limit the calculations to the spin-contaminated states and achieve purification through diagonalization of the effective Hamiltonian.

To simulate the spectra for the carbon and oxygen K-edge excitations of formaldehyde, we followed the procedure laid out in ref 52, i.e., we obtain difference densities from core–valence-separated TDA-TDDFT calculations (50 roots), where the occupied space is limited to the core orbital in question. We then compute orbital-optimized states, in which the constraint is only enforced on the α-spin sector, and include the resulting wave function and its spin-flipped analogue in the NOCI Hamiltonian. Calculations were performed using the aug-pcX-2 basis set,^128^ and the scaled scalar-relativistic zero-order regular approximation (sc-ZORA)^129–131^ with van Wüllen’s effective potential^132^ was used for the treatment of relativistic effects. Note that the ZORA Hamiltonian must also be included as part of the core Hamiltonian **h** in the energy expressions given in Table 1.

The resulting spectra are shown in Figure 10 alongside an experimental spectrum from ref 133, where we see excellent agreement of both COOX-based methods with the experimental spectrum. In direct comparison, the main-edge peak of the NOCI-COOX spectra appears to be slightly red-shifted versus the plain COOX calculations; otherwise, the simulated spectra are very closely matched. This high degree of accuracy is encouraging for future applications in the domain of core-excitations; in particular, NOCI-COOX may provide a pathway to excitations from higher shells (e.g., L-edge) with explicit computation of spin–orbit couplings between the reference states. Some care would have to be taken due to the nonorthogonality of the reference states, which would lead to a non-Hermitian Hamiltonian due to the spin–orbit coupling terms. A potential remedy could be the projection of the orbital-optimized wave functions onto the ground-state single-excitation manifold, which has been successfully applied for nonadiabatic dynamics simulations with ΔSCF,^134^ though other avenues should also be explored in future research.

**Figure 10:**
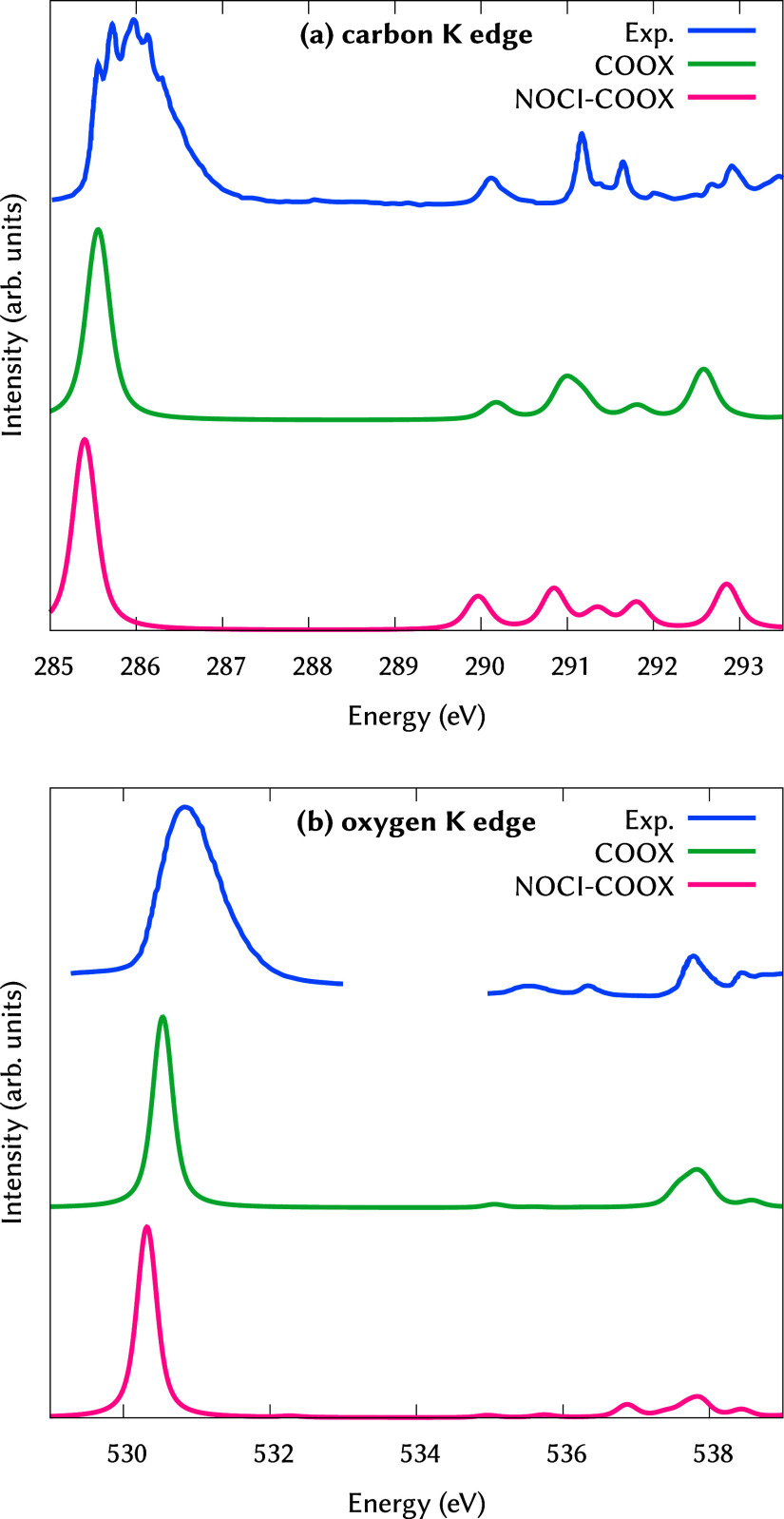
Near-edge X-ray absorption spectra of formaldehyde for excitation from the (a) carbon 1s and (b) oxygen 1s orbitals. Experimental spectrum adapted from ref 133, simulated spectra computed using COOX/PBE0/aug-pcX-2 and NOCI-COOX/PBE0/aug-pcX-2 and artificially broadened using Voigt profiles with a half-width-at-half-maximum (HWHM) of 0.15 eV for both the Gaussian and Lorentzian components.

## 5 Conclusion

Using only a moderate number of orbital-optimized reference states, the NOCI-COOX approach has been shown to deliver accurate and stable results for a range of chemically relevant challenging excited states such as states with substantial double-excitation character, conical intersections, and core excitations. Overall, ΔCOOX appears to be a more reliable source of orbital-optimized reference states as compared to IMOM or other ΔSCF-type approaches, which in many cases are plagued by convergence issues in the form of slow convergence, no convergence at all, or convergence to an undesired state, as well as numerical instability and the disappearance of reference states. Due to the more robust convergence of the underlying ΔCOOX method, NOCI-COOX also usually requires less computation time than an equivalent NOCI-IMOM calculation. Combined with our e-COOX embedding scheme, NOCI-COOX facilitates the treatment of excited states in complex molecular or bulk environments under consideration of the full quantum system, while the coupling to nonequilibrium polarizable continuum models is straightforward.

Performance-wise, NOCI-COOX, like other CI methods, falls into the category of “embarrassingly parallel” methods: Following the initial ground-state SCF calculation and selection of the excitation space, the individual ΔCOOX calculations and subsequent computations of matrix elements $H_{IJ}$ , $S_{IJ}$ , etc., can easily be distributed over multiple central processing units or compute nodes without data dependencies to accelerate computations.

These encouraging first results pave the way for a broad spectrum of future research directions for NOCI in general and NOCI-COOX in particular: The design of a DFT Hamiltonian beyond the simple weighted average of correlation corrections, as used in MS-DFT and in this work, might be of interest. Such a Hamiltonian could also include an explicit treatment of spin–orbit coupling to enable the full ab initio computation of core excitations beyond the K-edge. The implementation of more sophisticated spin-purification schemes^56,60^ could help to further minimize spin-contamination of the NOCI-COOX wave function and restore exact size-consistency for the NOCI-COOX energies. Furthermore, the availability of analytic nuclear gradients for the purposes of excited-state geometry optimizations and nonadiabatic molecular dynamics simulations would be a highly desirable supplement to the NOCI-COOX toolbox—to the best of our knowledge, analytic gradients have so far only been reported for the simplified cDFT-CI Hamiltonian of Van Voorhis and co-workers,^135^ and an extension to the Hamiltonians used in NOCI-COOX following the work of Mahler and Thompson^136^ should be investigated. A central area of potential future improvements is the numerical stability of the NOCI Hamiltonian for large reference spaces, where unphysical low-lying states can emerge if the SVD threshold is chosen to be too tight. Furthermore, while our simple orbital-energy-based selection criterion enables black-box-type applications of NOCI-COOX, a more thoughtful selection of reference states would be desirable to reduce the size of the NOCI Hamiltonian and thus the computational bottlenecking step without a loss of accuracy. Finally, although this work presented illustrative calculations from various corners of photochemistry, more comprehensive benchmarking is required to fully identify the strengths and weaknesses of NOCI-COOX.

## Supplementary Material





## Appendix A

### Derivation of $\left\langle {Ŝ}^{2}\right\rangle$

To derive $\left\langle {Ŝ}^{2}\right\rangle$ , we make use of the biorthogonal same-spin blocks of the transformed Slater determinants

A1
⟨φ̃iτI|φ̃jτJ⟩=σiτδij

where σ_*i*_^τ^ are the decreasingly ordered, non-negative singular values of the ττ-spin block of the MO overlap matrix. As a direct consequence of this representation, if there are any zeros in **σ** ^τ^, then they must appear as the last elements of **σ** ^τ^.

We use the ladder operator representation of ${Ŝ}^{2}$ , i.e.,

A2
$${Ŝ}^{2}={Ŝ}_{z}+{Ŝ}_{z}^{2}+{Ŝ}_{-}{Ŝ}_{+}$$

with the spin-lowering and -raising operators ${Ŝ}_{-}$ and ${Ŝ}_{+}$ , respectively. Since the NOCI Hamiltonian is block-diagonal with respect to the total spin of the wave function, we need only consider integrals over determinants with the same total spin and multiplicity, i.e., the same number of α- and β-electrons. Thus, integrals over ${Ŝ}_{z}$ and ${Ŝ}_{z}^{2}$ are easily evaluated as

A3
$$\left\langle \Phi_{I}\left|{Ŝ}_{z}\right|\Phi_{J}\right\rangle =\frac{\left\langle \Phi_{I}|\Phi_{J}\right\rangle }{2}\left(N_{\alpha }-N_{\beta }\right)$$

A4
$$\left\langle \Phi_{I}\left|{Ŝ}_{z}^{2}\right|\Phi_{J}\right\rangle =\frac{\left\langle \Phi_{I}|\Phi_{J}\right\rangle }{4}{\left(N_{\alpha }-N_{\beta }\right)}^{2}$$

where the overlap ⟨Φ_*I*_|Φ_*J*_⟩ is given by

A5
$$\left\langle \Phi_{I}|\Phi_{J}\right\rangle =\pm \prod_{i=1}^{N_{\alpha }}\sigma_{i}^{\alpha }\prod_{j=1}^{N_{\beta }}\sigma_{j}^{\beta }$$

For the same reasons, for the ladder operators, we get the simplification

A6
⟨ΦI|Ŝ−Ŝ+|ΦJ⟩=⟨ΦI|ΦJ⟩(Nβ−∑i=1Nα∑j=1Nβ⟨ϕ̃iαI|ϕ̃jβJ⟩⟨ϕ̃jβI|ϕ̃iαJ⟩σiασjβ)

where ϕ̃iαI denotes the spatial component of φ̃iαI . If ⟨Φ_*I*_|Φ_*J*_⟩ = 0, the final term in eq A6 does not vanish if there is at most one zero each in **σ** ^α^ and **σ** ^β^, as we illustrate in Table [Table tblA1].

**A1 tblA1:** Integrals $\left\langle \Phi_{I}\left|{Ŝ}_{-}{Ŝ}_{+}\right|\Phi_{J}\right\rangle$ for Different Numbers of Zeros in the Singular Values σ

zeros in **σ**	$\left\langle \Phi_{I}\left|{Ŝ}_{-}{Ŝ}_{+}\right|\Phi_{J}\right\rangle$
none	Equation A6
$\sigma_{N_{\alpha }}^{\alpha }$	∓∏i=1Nα−1σiα∏j=1Nβσjβ∑k=1Nβ⟨ϕ̃NααI|ϕ̃kβJ⟩⟨ϕ̃kβI|ϕ̃NααJ⟩σkβ
$$\sigma_{N_{\beta }}^{\beta }$$	analogous to $\sigma_{N_{\alpha }}^{\alpha }$
$\sigma_{N_{\alpha }}^{\alpha },\;\sigma_{N_{\beta }}^{\beta }$	∓∏i=1Nα−1σiα∏j=1Nβ−1σjβ⟨ϕ̃NααI|ϕ̃NββJ⟩⟨ϕ̃NββI|ϕ̃NααJ⟩
otherwise	0

## Notes

Excitation energies of polyenes and butadiene BLA scans for other functionals; butadiene difference densities for BHandHLYP reference states; IMOM and ΔCOOX energies for the butadiene BLA; stability analysis for the ethylene pyramidalization; and tabulated singlet and triplet energies corresponding to Table 3 (PDF)
Geometries in the *xyz* format (ZIP)
