# Supplementary material for: Nonorthogonal Configuration Interaction of Constraint-Based Orbital-Optimized Excited States: A Versatile Method for Theoretical Photochemistry
Source: J Chem Theory Comput. 2025 Oct 8;21(20):10193–211. doi: 10.1021/acs.jctc.5c01064 (PMC12573764; doi:10.1021/acs.jctc.5c01064)
Supplement: Supplementary file 1 [file ct5c01064_si_001.pdf]

Supporting Information

Non-Orthogonal Configuration Interaction of Constraint-Based Orbital-Optimized Excited States: A Versatile Method for Theoretical Photochemistry

Yannick Lemke,[†] Jörg Kussmann,[*,†] and Christian Ochsenfeld[*,†,‡]

†*Chair of Theoretical Chemistry, Department of Chemistry, Ludwig-Maximilians-Universität München, D-81377 Munich, Germany*

‡*Max-Planck-Institute for Solid State Research, D-70569 Stuttgart, Germany*

E-mail: joerg.kussmann@uni-muenchen.de; christian.ochsenfeld@uni-muenchen.de

Contents

1 $1\,^1\mathrm{B_u}$ and $2\,^1\mathrm{A_g}$ Energies for PBE and BHandHLYP Reference States 2

2 Butadiene Bond Length Alternation for Other Methods 4

3 Wavefunction Instability in the Ethylene Pyramidalization 9

4 Singlet and Triplet Energies of Butadiene, *E*-Hexatriene, and All-*E*-Octatetraene 9

References **11**

1 $1\,^1B_u$ and $2\,^1A_g$ Energies for PBE and BHandHLYP Reference States

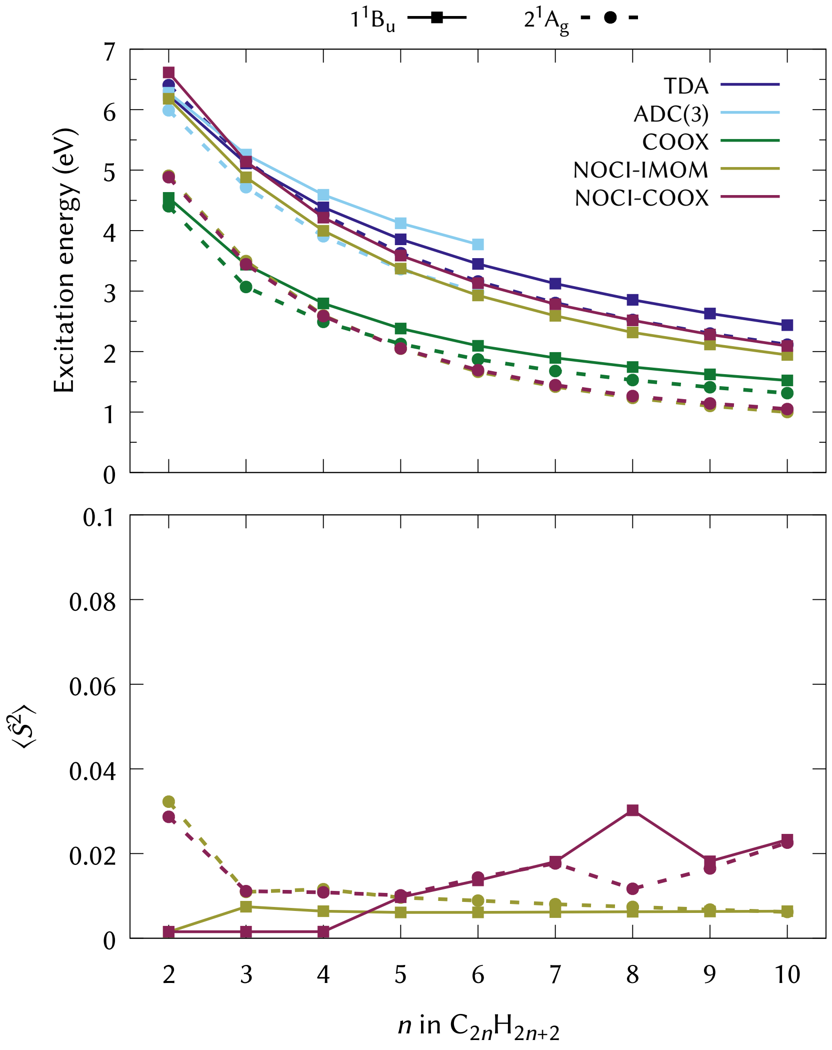

Figure S1: Vertical excitation energies and $\langle \hat{S}^2 \rangle$ values for the $1\,^1B_u$ and $2\,^1A_g$ states of linear polyenes $C_{2n}H_{2n+2}$ computed using ADC(3) and various methods at the PBE/def2-TZVP level (NOCI-IMOM/NOCI-COOX: 16/14 configurations, see Table 2 in the main text). Note that TDA, ADC(3), and plain COOX calculations are spin-restricted and thus all satisfy $\langle \hat{S}^2 \rangle = 0$.

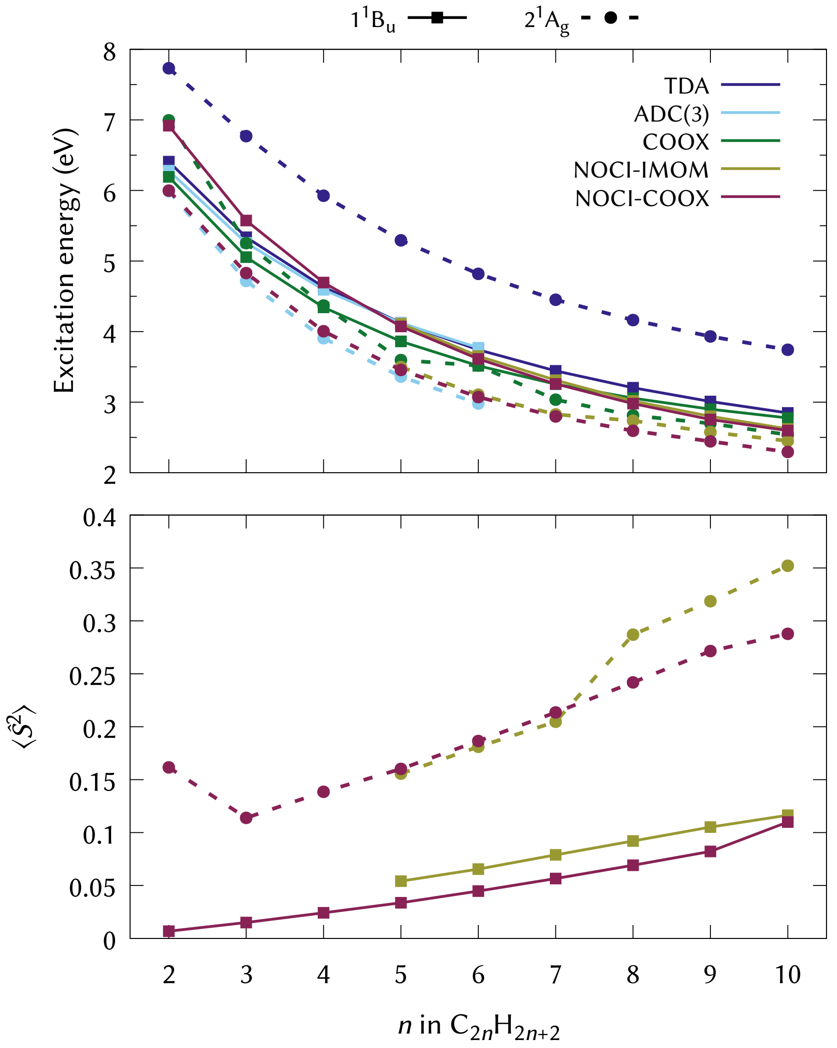

Figure S2: Vertical excitation energies and $\langle \hat{S}^2 \rangle$ values for the $1\,^1\mathrm{B_u}$ and $2\,^1\mathrm{A_g}$ states of linear polyenes $C_{2n}H_{2n+2}$ computed using ADC(3) and various methods at the BHandHLYP/def2-TZVP level (NOCI-IMOM/NOCI-COOX: 16/14 configurations, see Table 2 in the main text). Note that TDA, ADC(3), and plain COOX calculations are spin-restricted and thus all satisfy $\langle \hat{S}^2 \rangle = 0$. NOCI-IMOM values for $n = 2, 3, 4$ missing due to convergence issues.

2 Butadiene Bond Length Alternation for Other Methods

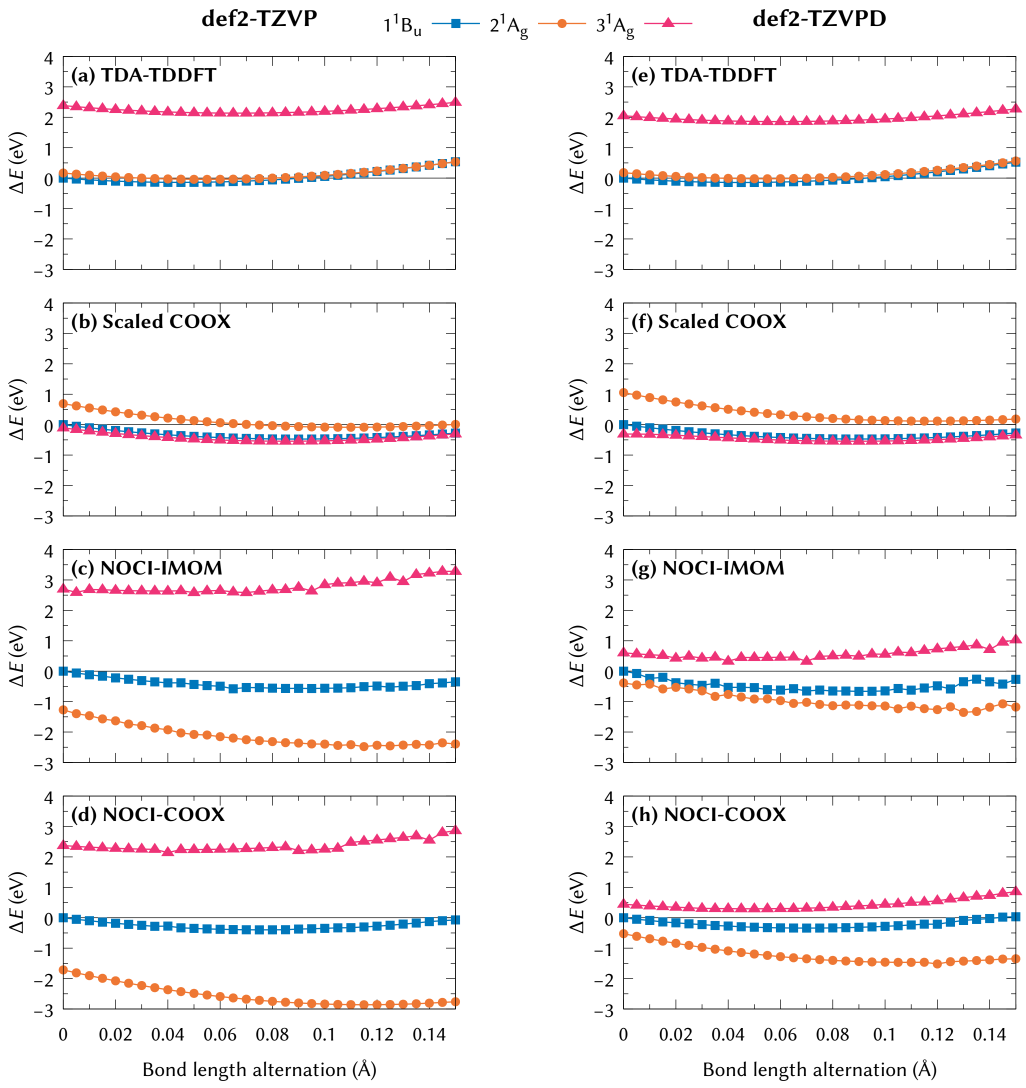

Figure S3: Bond length alternation of butadiene computed using PBE and the def2-TZVP (a–d) and def2-TZVPD (e–h) basis sets (NOCI-IMOM/NOCI-COOX: 16/14 configurations, see Table 2 in the main text). Energies relative to the $1\,^1B_u$ energy at the reference geometry.

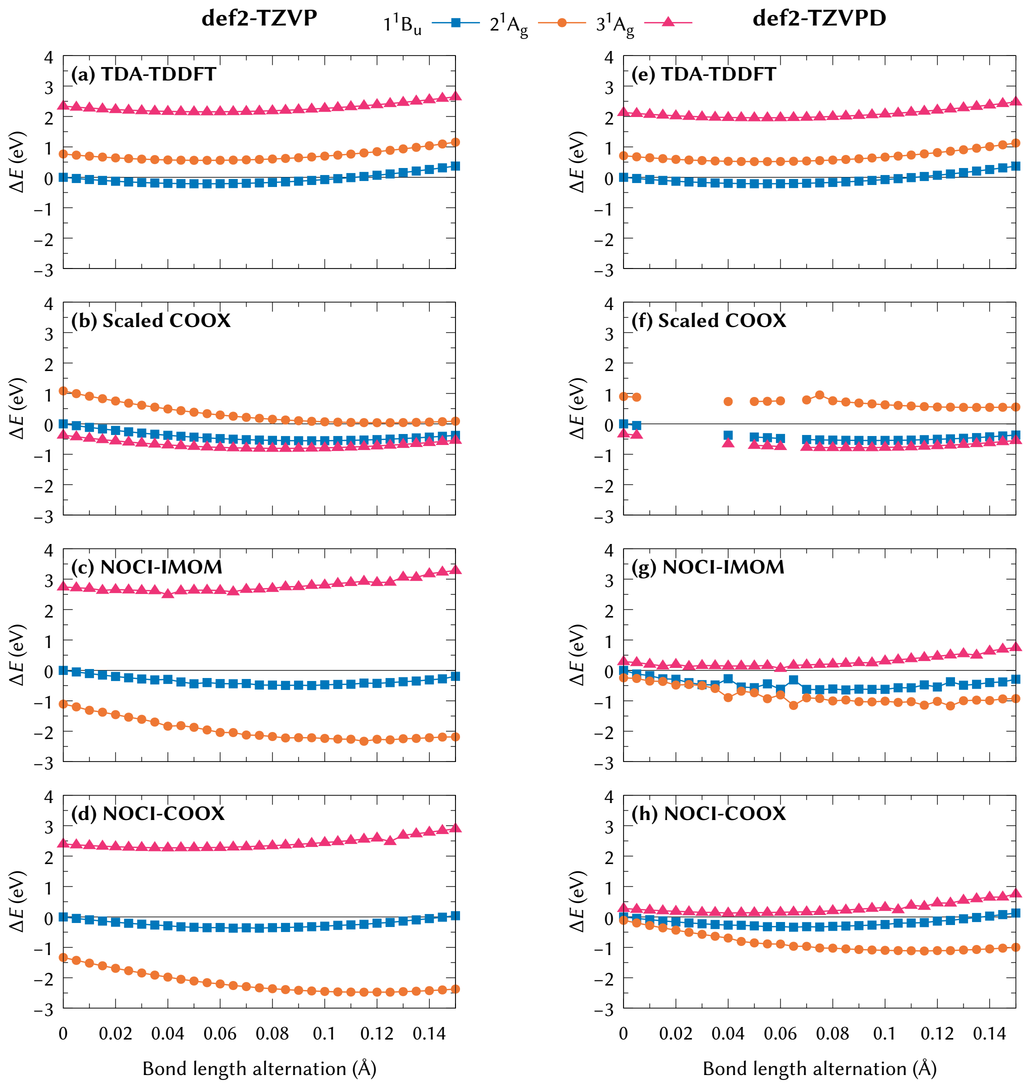

Figure S4: Bond length alternation of butadiene computed using PBE0 and the def2-TZVP (a–d) and def2-TZVPD (e–h) basis sets (NOCI-IMOM/NOCI-COOX: 16/14 configurations, see Table 2 in the main text). Energies relative to the $1\,^1B_u$ energy at the reference geometry.

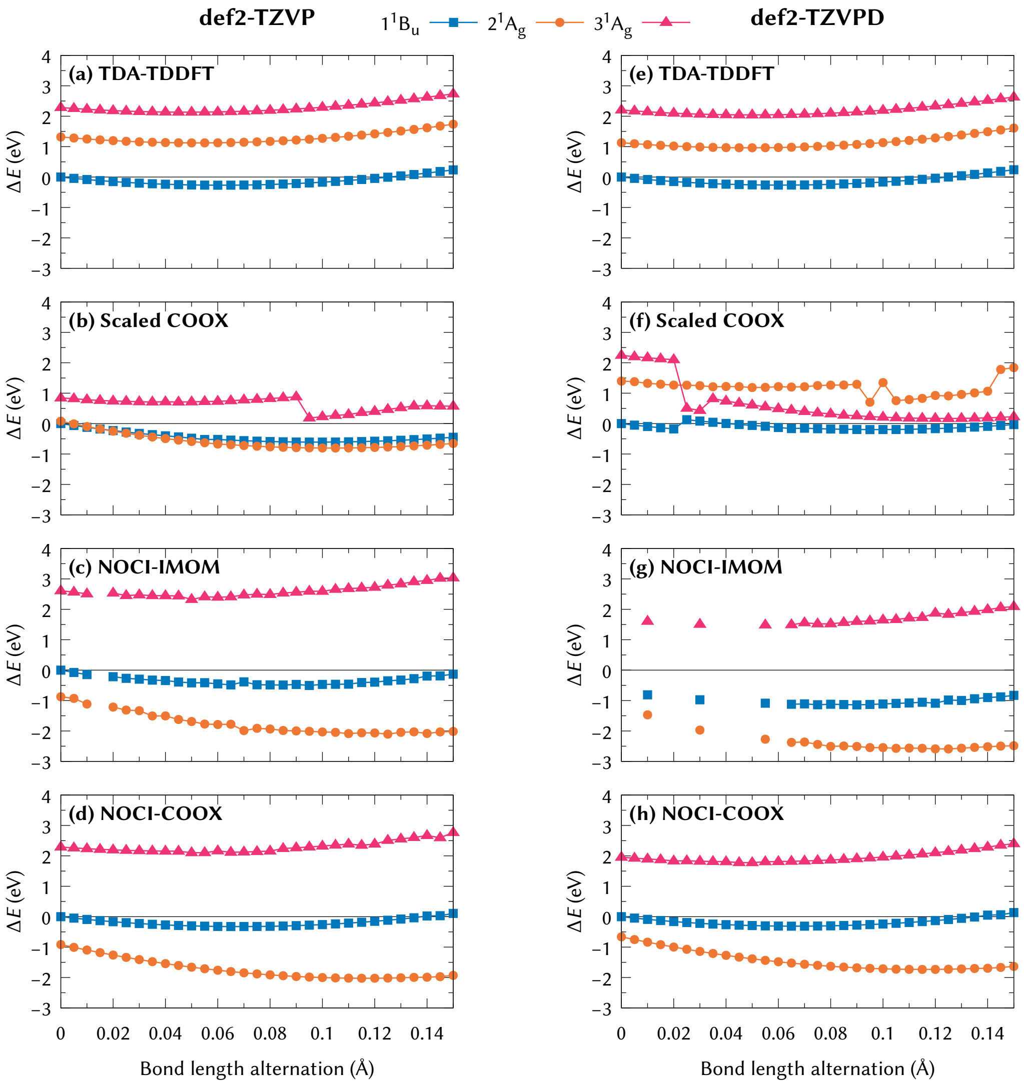

Figure S5: Bond length alternation of butadiene computed using BHandHLYP and the def2-TZVP (a–d) and def2-TZVPD (e–h) basis sets (NOCI-IMOM/NOCI-COOX: 16/14 configurations, see Table 2 in the main text). Energies relative to the $1\,^1B_u$ energy at the reference geometry.

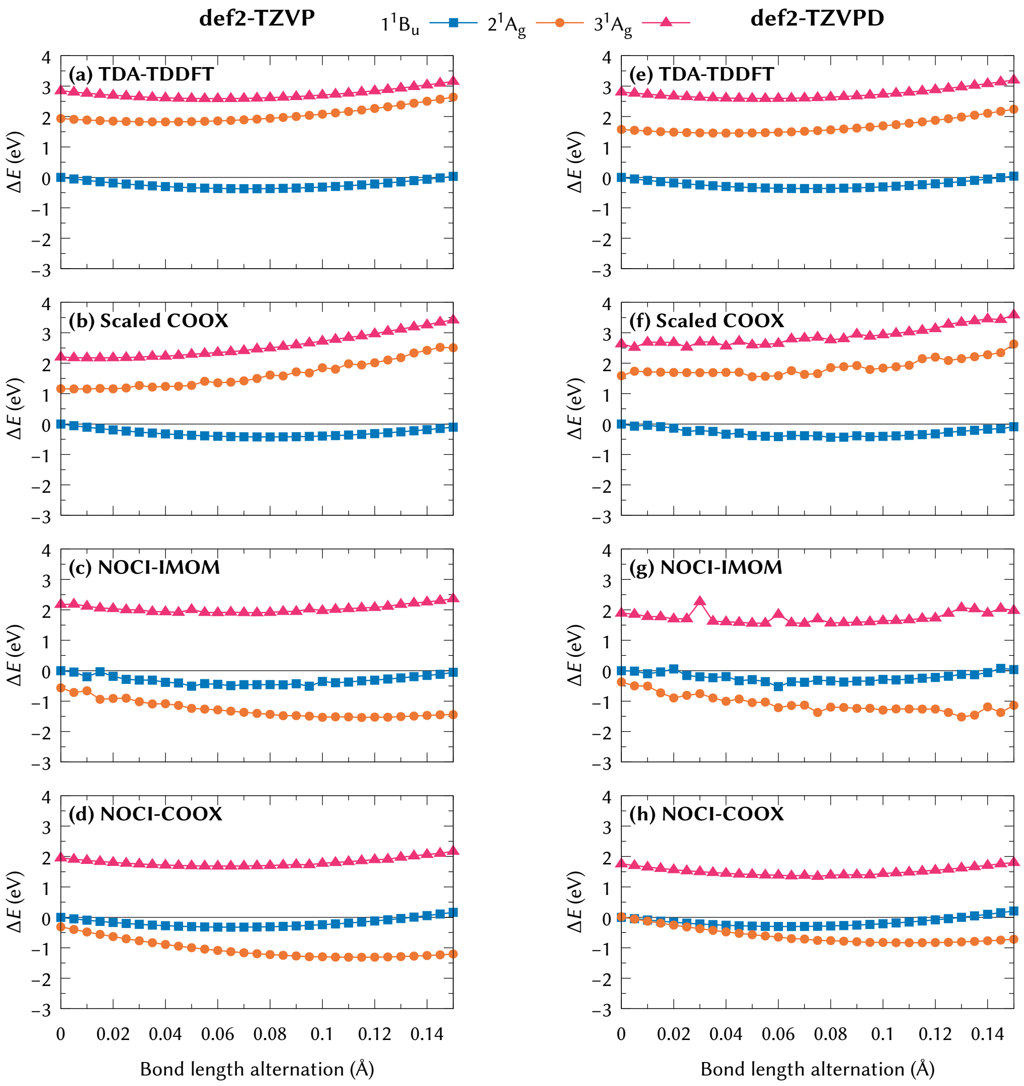

Figure S6: Bond length alternation of butadiene computed using HF and the def2-TZVP (a–d) and def2-TZVPD (e–h) basis sets (NOCI-IMOM/NOCI-COOX: 16/14 configurations, see Table 2 in the main text). Energies relative to the $1\,^1B_u$ energy at the reference geometry.

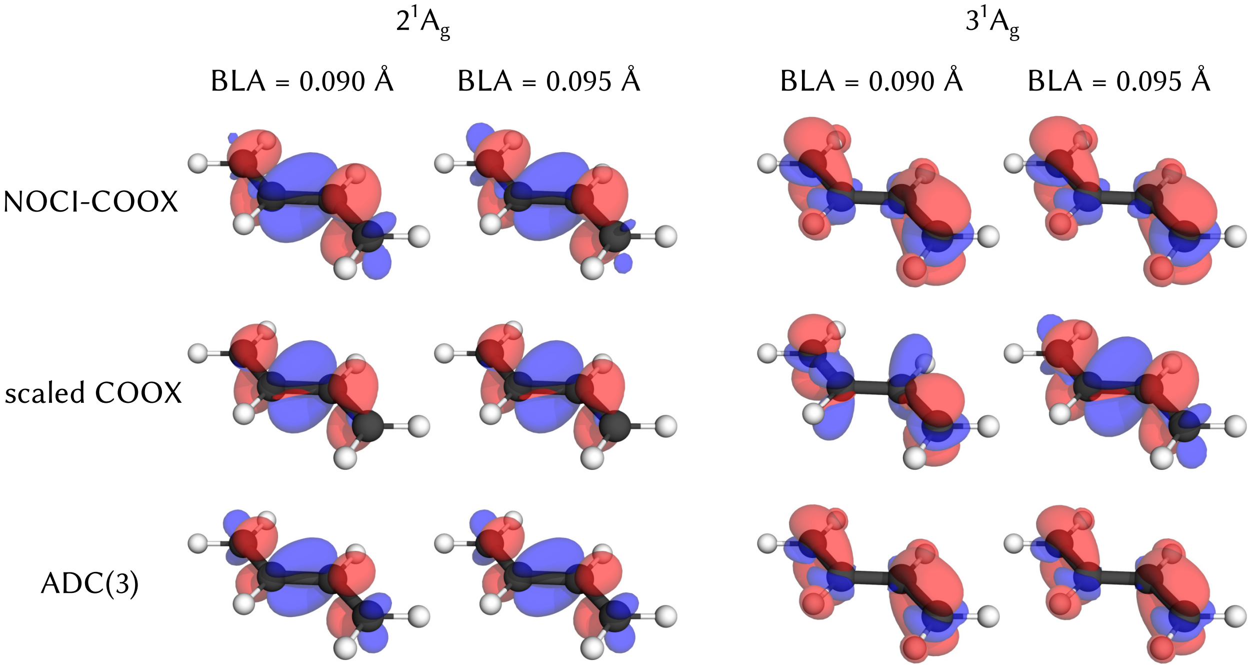

Figure S7: $2\,^1A_g$ and $3\,^1A_g$ difference densities for NOCI-COOX/BHandHLYP/def2-TZVP, scaled COOX/BHandHLYP/def2-TZVP, and ADC(3)/def2-TZVP for butadiene bond length alternations of 0.090 Å and 0.095 Å. For scaled COOX, the excited state character of the $3\,^1A_g$ state changes, coinciding with the energy discontinuity in Fig. S5(b).

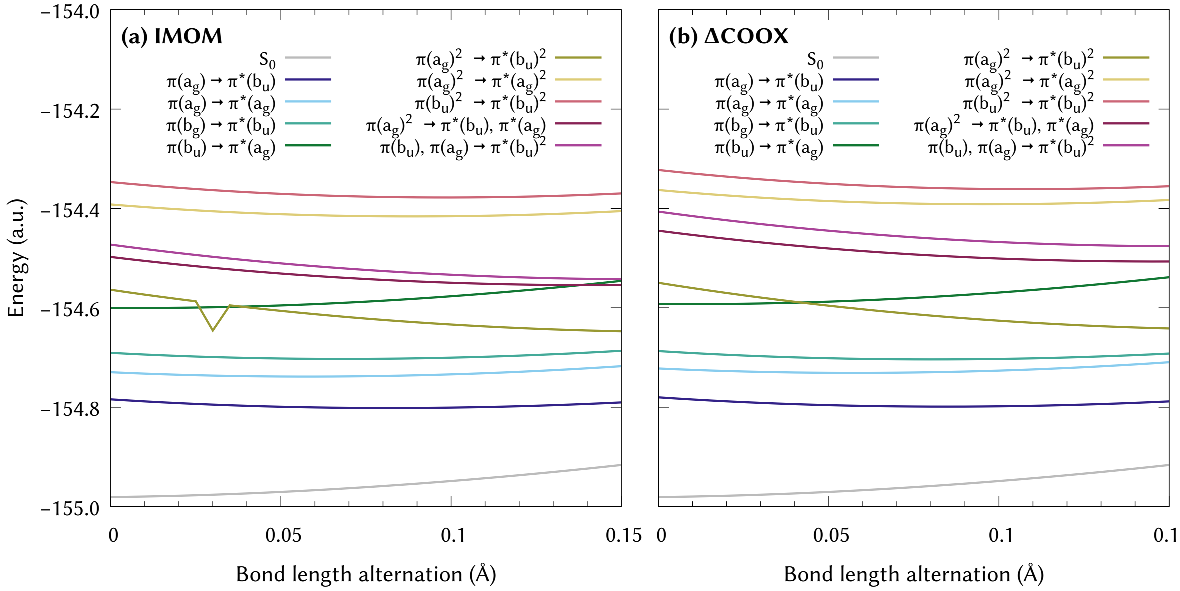

Figure S8: Energies of the orbital-optimized reference states for the butadiene bond length alternation corresponding to Fig. 6 in the main text, computed at the HF/def2-TZVPD level.

3 Wavefunction Instability in the Ethylene Pyramidalization

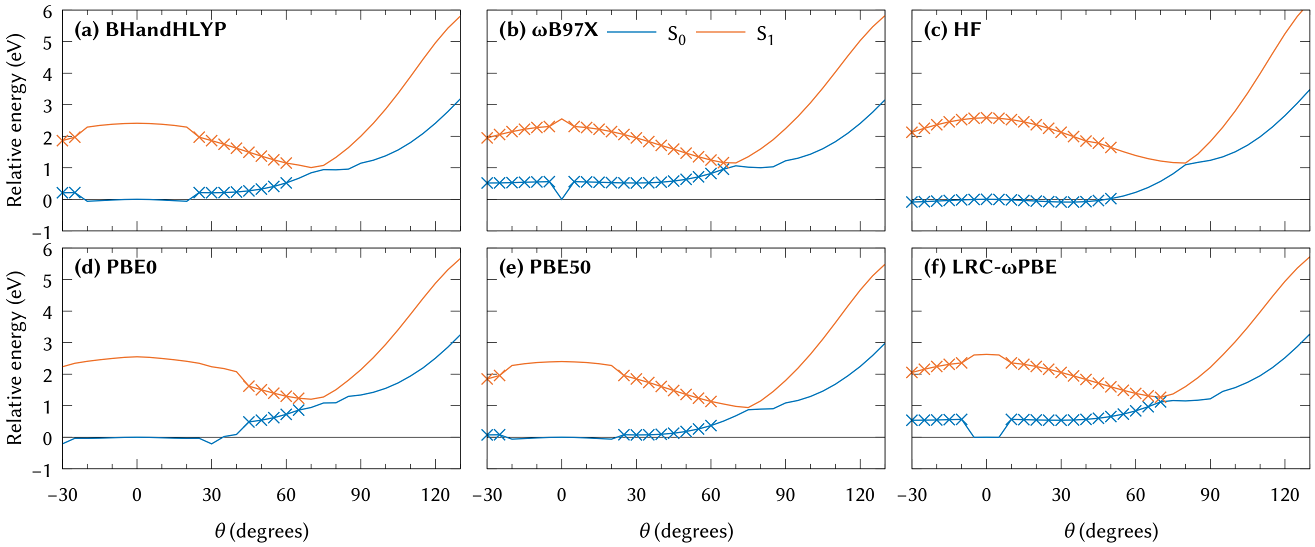

Figure S9: Relative NOCI-COOX/def2-TZVP energies for the ethylene pyramidalization computed using different functionals. For each geometry, the initial guess is constructed as a superposition of atomic densities. For points labelled ×, the Kohn–Sham ground-state wavefunction has an RHF/RHF instability.

4 Singlet and Triplet Energies of Butadiene, *E*-Hexatriene, and All-*E*-Octatetraene

Table S1: Vertical excitation energies in eV for the lowest-lying singlet and triplet states of butadiene, E-hexatriene, and all-E-octatetraene.

method	butadiene				E-hexatriene				all-E-octatetraene			
	$1\,^1B_u$	$2\,^1A_g$	$1\,^3B_u$	$1\,^3A_g$	$1\,^1B_u$	$2\,^1A_g$	$1\,^3B_u$	$1\,^3A_g$	$1\,^1B_u$	$2\,^1A_g$	$1\,^3B_u$	$1\,^3A_g$
**reference**												
TBE[a]	6.18	6.55	3.20	5.08	5.10	5.09	2.40	4.15	4.66	4.47	2.20	3.55
CASPT2[b]	6.38	6.43	3.30	5.08	5.18	5.33	2.67	4.22	4.35	4.52	2.27	3.61
DFT/MRCI[c]	6.02	6.18	3.09	4.85	4.95	4.92	2.45	3.96	4.25	4.01	2.04	3.32
ADC(3)[d]	6.21	5.83	3.06	4.92	5.21	4.59	2.40	4.03	4.55	3.80	2.00	3.36
**NOCI-COOX**[e]												
SVWN5	6.53	4.90	1.73	3.33	5.10	3.44	1.09	2.50	4.18	2.58	0.77	1.87
PBE	6.50	4.81	1.74	3.29	5.08	3.38	1.09	2.45	4.17	2.53	0.77	1.83
PBE0	6.64	5.38	2.28	3.87	5.28	4.06	1.64	3.08	4.39	3.22	1.32	2.47
PBE50	6.81	5.98	2.84	4.48	5.51	4.79	2.20	3.77	4.65	3.96	1.88	3.17
LRC-ωPBE	6.92	5.86	2.73	4.25	5.69	4.91	2.26	3.86	4.87	4.23	2.05	3.42
LRC-ωPBEh	6.81	5.72	2.61	4.15	5.56	4.67	2.10	3.62	4.74	3.97	1.87	3.15
BHandHLYP	6.75	6.00	2.87	4.54	5.48	4.81	2.21	3.78	4.63	3.97	1.88	3.18
ωB97X	7.00	6.13	2.96	4.55	5.77	5.16	2.46	4.10	4.95	4.47	2.22	3.63
HF	6.95	6.90	4.02	5.73	5.85	6.21	3.37	5.24	5.09	5.41	3.03	4.76
**NOCI-IMOM**[e]												
SVWN5	6.12	4.97	1.69	3.33	4.82	3.51	1.04	2.31	3.95	2.61	0.73	1.69
PBE	6.09	4.82	1.67	3.23	4.82	3.43	1.00	2.29	3.96	2.54	0.71	1.66
PBE0	6.36	5.27	2.09	3.65	5.20	4.12	1.39	3.08	4.33	3.25	1.15	2.43
PBE50		n.c.[f]				n.c.[f]				n.c.[f]		
LRC-ωPBE	6.69	5.44	2.21	3.72		n.c.[f]				n.c.[f]		
LRC-ωPBEh	6.55	5.41	2.20	3.70		n.c.[f]			4.72	4.06	1.46	3.02
BHandHLYP		n.c.[f]				n.c.[f]			4.67	3.96	1.57	3.19
ωB97X		n.c.[f]				n.c.[f]				n.c.[f]		
HF	6.97	6.53	3.54	5.29	6.00	6.09	2.74	5.46		n.c.[f]		

[a] Theoretical best estimates from refs 1,2. [b] aug-cc-pVTZ basis set, data from ref 2. [c] Ahlrichs TZVP basis set, data from ref 1. [d] def2-TZVP basis set, this work. [e] def2-TZVPD basis set, reference states as defined in Table 2 in the main text. [f] IMOM calculations did not converge within 500 iterations.

References

(1) Silva-Junior, M. R.; Schreiber, M.; Sauer, S. P. A.; Thiel, W. Benchmarks for electronically excited states: Time-dependent density functional theory and density functional theory based multireference configuration interaction. *J. Chem. Phys.* **2008**, *129*, 104103.

(2) Silva-Junior, M. R.; Schreiber, M.; Sauer, S. P. A.; Thiel, W. Benchmarks of electronically excited states: Basis set effects on CASPT2 results. *J. Chem. Phys.* **2010**, *133*, 174318.
